# Penicillin induces alterations in glutamine metabolism in *Streptococcus pneumoniae*

**DOI:** 10.1038/s41598-017-15035-y

**Published:** 2017-11-06

**Authors:** Jessica Y. El Khoury, Nancy Boucher, Michel G. Bergeron, Philippe Leprohon, Marc Ouellette

**Affiliations:** Centre de Recherche en Infectiologie du Centre de Recherche du CHU de Québec and Département de Microbiologie, Infectiologie et Immunologie, Faculté de Médecine, Université Laval, Québec, Québec, Canada

## Abstract

Penicillin is a bactericidal antibiotic that inhibits the synthesis of the peptidoglycan by targeting penicillin-binding proteins. This study aimed to assess through transcriptional profiling the stress response of *S*. *pneumoniae* strains after exposure to lethal penicillin concentrations to understand further the mode of action of penicillin. Two experimental designs (time-course and dose-response) were used for monitoring the effect of penicillin on the transcriptional profile. The expression of some genes previously shown to be modulated by penicillin was altered, including *ciaRH*, *pstS* and *clpL*. Genes of the *glnRA* and *glnPQ* operons were among the most downregulated genes in the three strains. These genes are involved in glutamine synthesis and uptake and LC-MS work confirmed that penicillin treatment increases the intracellular glutamine concentrations. Glutamine conferred a protective role against penicillin when added to the culture medium. Glutamine synthetase encoded by *glnA* catalyses the transformation of glutamate and ammonium into glutamine and its chemical inhibition by the inhibitor L-methionine sulfoximine is shown to sensitize *S*. *pneumoniae* to penicillin, including penicillin-resistant clinical isolates. In summary, a combination of RNA-seq and metabolomics revealed that penicillin interferes with glutamine metabolism suggesting strategies that could eventually be exploited for combination therapy or for reversal of resistance.

## Introduction

*Streptococcus pneumoniae* is a Gram-positive bacteria responsible for several diseases such as otitis, sinusitis, pneumonia, sepsis and meningitis^1^. People at risk are the elderly and young children, pneumococcal infections being responsible for the death of about 393 000 children under the age of five every year^2^. Over the past decades, antibiotic resistance among *S*. *pneumoniae* has steadily increased, with 15 to 30% of the strains currently being classified as multi-resistant^3^. The introduction of two pneumococcal conjugate vaccines (PCV7 and PCV13) has slowed the spread of several epidemiologically significant serotypes, but antibiotic resistant rates continue to rise^4,5^.

Penicillin (PEN), a β-lactam antibiotic, remains a pillar against pneumococcal infections but resistant clones worldwide threaten its efficacy. This is especially true for pneumococcal meningitis against which third generation cephalosporins became the recommended treatment for non-susceptible PEN strains^6^. PEN is a bactericidal antibiotic inhibiting the synthesis of the peptidoglycan layer of the bacterial cell wall by targeting penicillin binding proteins (PBPs). PBPs are membrane-associated enzymes involved in the final steps of peptidoglycan assembly^7^. However, small molecules can have pleiotropic effects on cells and understanding the precise molecular events responsible for drug-induced events holds the promise of improving current therapies^8^. Nucleic acids sequencing, whether DNA or RNA has helped in detecting effector mutations linked to antibiotics mode of action and this has been applied to *S*. *pneumoniae*. Indeed, sequencing the genome of β-lactam resistant *S*. *pneumoniae* confirmed the role of PBPs in resistance^9–12^ but in addition also highlighted novel pathways and genes necessary for β-lactam resistance^13,14^.

Gene expression modulation, either at the transcriptional or translational levels, plays a central role in cellular adaptation to stresses^15^. Gene expression profiling is thus well suited for deciphering the mode of action of drugs given that expression alteration usually mirrors the cellular response to drug-induced damage^16,17^. For example, a microarray-based gene expression analysis in *S*. *pneumoniae* showed that the fluoroquinolone levofloxacin induces an upregulation of the *fatDCEB* operon coding for an iron transporter, leading to increased intracellular iron concentrations and ultimately to the accumulation of ROS^18^. Other microarray-based studies in *S*. *pneumoniae* revealed many genes whose expression is modulated in response to different antibiotics but did not assess their outcome on resistance or susceptibility^19–21^. Thus, a better understanding of the mode of action of PEN and of the cellular response induced by it would reveal the extent of metabolic alterations associated with cell death and identify new strategies to improve its effectiveness. Whole RNA sequencing is now surpassing DNA microarrays although not yet reported for studying β-lactam resistance or mode of action. However RNA-seq was successfully used in *S*. *pneumoniae* to look at the role of small RNAs on virulence^22^, on host-pathogen interactions^23,24^ or at tetracycline resistance in *S*. *pneumoniae* ^25^.

We performed here a detailed RNA-seq experiment of *S*. *pneumoniae* when subjected to penicillin. We discovered that glutamine metabolism is altered and that interfering with glutamine synthesis can alter susceptibility to penicillin.

## Results

### Transcriptional response of *S*. *pneumoniae* to penicillin

Two experimental designs were used to monitor the effect of PEN on the transcriptional profile of *S*. *pneumoniae* R6. The first design analysed gene expression over time in *S*. *pneumoniae* R6 exposed to PEN at its minimum inhibitory concentration (MIC) and in control cells grown in parallel in the absence of antibiotic. Because understanding metabolic alterations requires working with cells that are still metabolically active, gene expression was monitored at three time points corresponding to survival rates of ~85% (T1), ~70% (T2) and ~50% (T3) (Supplementary Fig. S1b), in addition to the T0 baseline. All time points were selected to fit into the *S*. *pneumoniae* R6 logarithmic phase of growth to minimize biases coming from growth-related regulation of gene expression. Only genes with a fold change (FC) higher than 2 (log_2_ FC ≤ −1 or log_2_ FC ≥ +1) and with a false discovery rate adjusted *p* value ≤ 0.05 (*q*-value) compared to the T0 baseline were considered as significantly modulated. Overall, 105 genes were differentially expressed upon exposure to PEN, of which 48 were overexpressed and 57 were downregulated (Table 1). Among the genes steadily upregulated over time were a number of molecular chaperones (spr0453–0456), metabolic enzymes (e.g. spr1074–1075) and uncharacterized proteins, as well as the two component regulator *ciaRH* (spr0707 and spr0708) already documented to be overexpressed upon PEN exposure^19^. The CiaRH two component system is known to repress the competence regulon^26^ and many competence-related genes had indeed their expression decreased from T0 to T3 (Table 1). These include the competence protein coding genes *celB* (spr0857), the operon *cglA*, *cglB*, *cglC* and *cglD* (spr1861–1864), the competence-specific transcription regulators *comX1* and *comX2* (spr0013, spr1819) and the single-stranded DNA-binding protein *ssbB* (spr1724) (Table 1). Genes coding for a phosphate ABC permease previously linked to PEN resistance was also overexpressed (spr1896–1899 in Table 1). Interestingly, the expression of several genes involved in glutamine metabolism steadily decreased upon PEN exposure (spr0443, spr0444 and spr1121 in Table 1). These are coding for the ABC transporter GlnQ, the transcriptional regulator GlnR and the glutamine synthetase GlnA, the latter two genes being the most downregulated from the dataset (Table 1).

**Table 1:** Genes modulated by PEN in *S*. *pneumoniae* R6 in the time-course transcriptomic design.

Entry no. in R6 genome database	Gene Symbol	Gene description	Log_2_FC (*q*-Value ≤ 0.05)^a,b^					
			No PEN^c^			PEN at 1 X MIC^d^		
			T1/T0	T2/T0	T3/T0	T1/T0	T2/T0	T3/T0
**Downregulated**								
spr0013	*comX1*	competence- specific global transcription modulator	—	—	—	—	−1.22	—
spr0020	—	hypothetical protein	—	—	—	—	−1.68	−2.42
spr0024	—	hypothetical protein	—	—	—	—	—	−1.04
spr0041	*IS1167*	transposase	—	—	—	—	−1.17	—
spr0042	*IS1167*	transposase	—	—	—	—	−1.22	—
spr0117	—	hypothetical protein	—	—	—	—	−1.22	—
spr0120	—	hypothetical protein	—	—	—	−1.32	—	—
spr0123	—	MutT/nudix family protein	—	—	—	—	—	−1.55
spr0127	*orf51*	hypothetical protein	—	−1.23	−1.23	−1.00	−1.74	−3.00
spr0128	—	hypothetical protein	—	—	—	—	−1.58	−2.26
spr0210	*adk*	adenylate kinase	—	—	—	—	—	−1.35
spr0352a	—	DNA- binding protein	−2.00	−1.00	−1.00	−2.00	−2.00	—
spr0379	*fabK*	enoyl- acyl carrier protein(ACP) reductase	—	—	—	—	—	−1.14
spr0387	*accA*	acetyl- CoA carboxylase subunit alpha	—	—	—	−1.09	—	—
spr0388	—	hypothetical protein	−1.31	—	—	−1.33	−1.36	−1.91
spr0432	*cspR*	rRNA methylase	—	—	—	—	−1.14	−1.87
**spr0443**	***glnR*** ^**e**^	**transcriptional repressor of the glutamine synthetase gene**	**—**	**—**	**—**	**—**	**−1.79**	**−3.37**
**spr0444**	***glnA*** ^**e**^	**glutamine synthetase, type I**	**—**	**—**	**—**	**—**	**−1.62**	**−3.02**
spr0445	*hsdS*	type I restriction- modification system S subunit	—	—	—	—	−1.27	−2.05
spr0446	*hsdS*	type I restriction- modification system S subunit	—	—	—	—	−1.49	−2.37
spr0480	—	hypothetical protein	—	—	—	—	—	−1.00
spr0499a	—	hypothetical protein	—	−1.00	−1.00	−1.00	—	—
spr0504	*licT*	BglG family transcriptional antiterminator	—	−1.58	−2.43	—	—	−1.05
spr0560a	—	hypothetical protein	—	—	—	−1.17	−2.17	—
spr0629	*thiM*	hydroxyethylthiazole kinase	—	—	—	—	—	−1.00
spr0683	—	hypothetical protein	—	—	—	—	—	−1.25
spr0857	*celB*	competence protein CelB	—	—	—	—	−1.17	—
spr0936	*ABC-MSP*	iron- compound ABC transporter permease	—	—	—	—	—	−1.37
spr0940	—	hypothetical protein	—	—	—	—	−1.05	−1.76
spr0941	—	hypothetical protein	—	—	—	—	−1.32	−2.06
spr0942	*ccrB*	hypothetical protein	—	—	—	—		−1.26
spr0943	—	hypothetical protein	—	—	—	—	−1.03	−1.53
spr0988	*IS1167*	transposase	—	—	—	—	−1.16	−1.36
spr1064	*nrdH*	glutaredoxin- like protein	−1.28	—	—	—	−1.18	—
**spr1121**	***glnQ*** ^**e**^	**amino acid ABC transporter ATP- binding protein**	**—**	**—**	**—**	**—**	**−1.14**	**−1.83**
spr1144	*smf*	DNA processing protein DprA	−1.00	—	—	−1.42	−1.42	—
spr1155	*pyrB*	aspartate carbamoyltransferase	—	—	—	—	—	−1.30
spr1156	*pyrR*	bifunctional pyrimidine regulatory protein PyrR uracil phosphoribosyltransferase	—	—	—	—	—	−1.41
spr1208	—	hypothetical protein	—	—	—	—	—	−1.58
spr1210	—	hypothetical protein	—	—	—	−1.32	−1.32	−2.32
spr1316	—	hypothetical protein	—	—	—	—	−1.17	—
spr1409	—	glutathione S- transferase YghU	—	—	—	—	—	−1.11
spr1440	—	ATP- dependent RNA helicase	—	—	—	—	—	−1.11
spr1724	*ssbB*	single- stranded DNA- binding protein	—	—	—	—	−1.32	−1.74
spr1806	—	cell wall surface anchor family protein	—	—	—	—	−1.04	−1.49
spr1817	*ABC-NBD*	ABC transporter ATP- binding protein	—	—	—	—	−1.03	−1.67
spr1818	—	hypothetical protein	—	—	—	−1.03	−1.37	−2.46
spr1819	*comX2*	competence- specific global transcription modulator	—	—	—	—	−1.22	—
spr1858	—	hypothetical protein	—	—	−1.28	—	−1.50	—
spr1859	—	hypothetical protein	—	—	—	—	−1.00	—
spr1861	*cglD*	competence protein CglD	—	—	—	−1.16	−1.38	−2.16
spr1862	*cglC*	competence protein CglC	—	−1.14	−1.14	−1.46	−1.87	−2.14
spr1863	*cglB*	competence protein CglB	—	—	−1.00	−1.00	−1.49	−1.81
spr1864	*cglA*	competence protein CglA	—	—	—	−1.00	−1.26	—
spr1993	*hslO*	Hsp33- like chaperonin	—	—	—	—	—	−1.19
spr2023	*mreC*	rod shape- determining protein MreC	—	—	—	—	—	−1.19
spr2024	*ABC-MSP*	ABC transporter permease	—	—	—	—	—	−1.07
**Upregulated**								
spr0096	—	LysM domain-containing protein	—	—	—	—	1.46	1.84
spr0262	*adhP*	alcohol dehydrogenase	—	—	—	—	—	1.17
spr0287	*kdgA*	keto-hydroxyglutarate-aldolase/keto-deoxy-phosphogluconate aldolase	1.00	—	1.00	—	1.58	—
spr0289	—	hypothetical protein	1.00	—	—	1.00	1.58	—
spr0307	*clpL*	ATP-dependent protease ATP-binding subunit	—	—	—	—	—	2.16
spr0311	—	hypothetical protein	—	—	—	1.32	—	2.04
spr0342	—	hypothetical protein	—	—	—	—	—	1.25
spr0343	*hk03*	sensor histidine kinase	—	—	—	—	—	1.29
spr0441	*pgk*	phosphoglycerate kinase	—	1.11	1.26	1.11	1.18	—
spr0453	*hrcA*	heat-inducible transcription repressor	1.31	—	—	1.36	1.87	—
spr0454	*grpE*	heat shock protein GrpE	—	—	—	1.23	1.64	1.36
spr0455	*dnaK*	molecular chaperone DnaK	1.34	—	1.08	1.80	2.24	2.07
spr0456	*dnaJ*	molecular chaperone DnaJ	1.40	1.00	1.03	1.57	1.91	1.73
spr0471	—	hypothetical protein	—	1.28	1.10	—	1.00	—
spr0562	*PTS-EII*	PTS system transporter subunit IIA	—	—	—	—	—	1.54
spr0563	—	hypothetical protein	—	—	—	—	—	1.22
spr0633	—	hypothetical protein	—	—	—	—	1.00	—
spr0635	—	hypothetical protein	—	—	—	—	2.00	—
spr0707	*ciaR*	DNA-binding response regulator CiaR	—	—	—	—	1.24	1.81
spr0708	*ciaH*	sensor histidine kinase CiaH	—	—	—	—	1.52	2.09
spr0782	—	hypothetical protein	—	—	—	1.67	2.66	3.63
spr0810	—	hypothetical protein	—	—	—	—	1.59	2.27
spr0884	*prsA*	foldase PrsA	—	—	—	—	1.30	1.63
spr0931	*-*	hypothetical protein	—	—	—	—	—	1.94
spr0959	—	hypothetical protein	—	-	—	—	2.00	3.00
spr0997	—	hypothetical protein	—	—	—	—	—	1.15
spr1028	*gapN*	glyceraldehyde-3-phosphate dehydrogenase	1.34	1.04	1.00	—	—	1.20
spr1074	*lacC*	tagatose-6-phosphate kinase	—	—	—	—	—	1.17
spr1075	*lacB*	galactose-6-phosphate isomerase subunit LacB	—	—	—	1.08	1.35	1.30
spr1097	*nirC*	formate/nitrate transporter	—	1.32	1.22	1.50	1.94	3.17
spr1293	*ABC-NBD*	ABC transporter ATP-binding protein	—	—	—	—	—	2.81
spr1536	*nanA*	neuraminidase A	—	—	—	—	—	3.00
spr1538	*axe1*	acetyl xylan esterase	—	—	—	—	1.70	2.65
spr1683	—	NAD-dependent epimerase/dehydratase	—	—	—	—	1.32	2.46
spr1700	*treR*	trehalose operon transcriptional repressor	——	—	—	1.28	—	—
spr1722	*groEL*	molecular chaperone GroEL	—	—	—	—	1.49	—
spr1800	—	hypothetical protein	1.00	—	—	1.00	—	—
spr1837	*adhE*	bifunctional acetaldehyde-CoA/alcohol dehydrogenase	2.56	2.43	2.57	2.28	3.05	3.45
spr1866	*adh*	zinc-containing alcohol dehydrogenase	—	—	1.03	1.06	—	—
spr1875	—	hypothetical protein	—	—	—	—	1.71	2.45
spr1895	*pstS*	phosphate ABC transporter substrate + B766-binding protein	—	—	—	1.24	—	—
spr1896	*pstC*	phosphate ABC transporter permease	—	—	—	1.42	—	—
spr1898	*pstB*	phosphate transporter ATP-binding protein	—	—	—	1.21	—	—
spr1899	*phoU*	phosphate transporter PhoU	—	—	—	1.58	—	—
spr1916	*malP*	maltodextrin phosphorylase	—	—	—	—	1.33	2.12
spr2011	—	ribosomal subunit interface protein	—	—	—	—	1.40	1.94
spr2045	*sphtra*	serine protease	—	—	—	—	1.49	1.97
spr2046	*spo0J*	chromosome segregation protein	—	—	—	—	1.64	2.09

^a^PEN was added at 1 X MIC at T0 and the differential gene expression was tested for three time points (T1, T2 and T3). Genes included showed significant variations of log_2_ FC ≤ −1 or log_2_ FC ≥ +1 with a *q*-value ≤ 0.05.

^**b**^ ‘-’ means no significant change in expression.

^**c**^‘No PEN’ corresponds to the genes modulated in the untreated R6.

^**d**^‘PEN’ corresponds to the genes modulated in the treated R6.

^**e**^Genes involved in glutamine metabolism are shown in bold.

**Figure 1:**
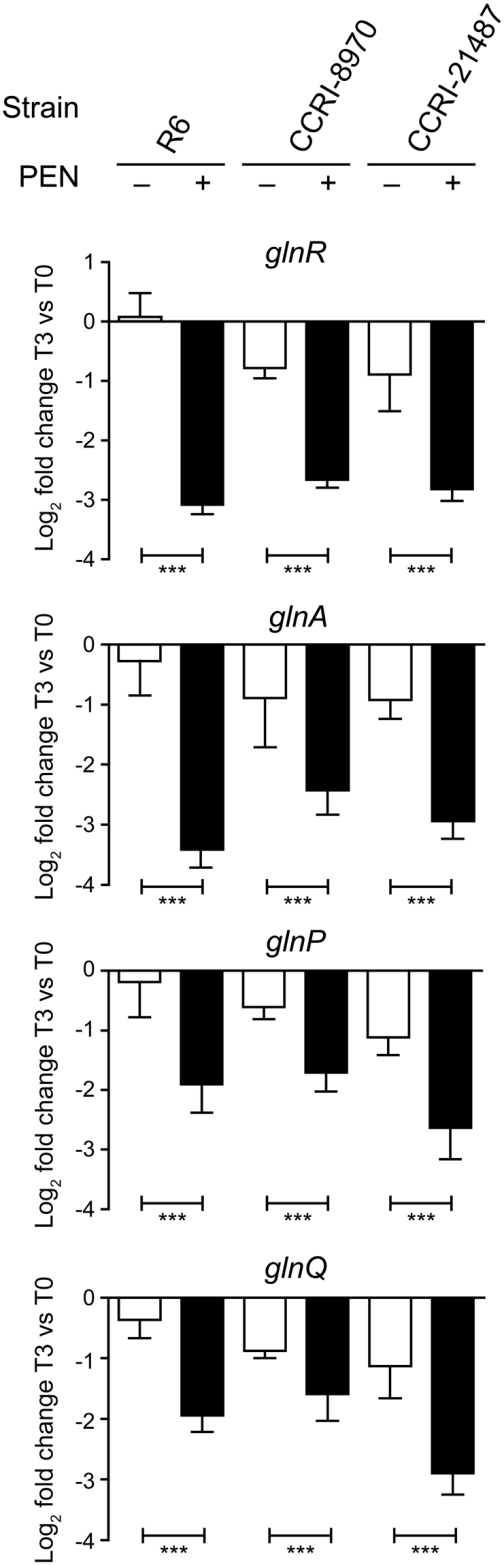
Validation of penicillin-induced alterations to glutamine metabolism gene expression in *S*. *pneumoniae* by qRT-PCR. Genes involved in glutamine metabolism (*glnR*, *glnA*, *glnP* and *glnQ*) found to be down-regulated after exposure to PEN by RNA-seq were validated by qRT-PCR. RNA levels were normalized based on the amplification signals of 16S ribosomal RNA. Graphs show the log_2_ fold change of expression at time T3 (corresponding to 40 min for R6, 28 min for CCRI-21487 and 85 min for CCRI-8970) over T0 in untreated (white bars) and PEN-treated (black bars) *S*. *pneumoniae* isolates. Results are displayed as mean ± SD of three biological replicates and significant differences are identified as determined by split plot design and Fisher’s F-test (***p ≤ 0.001).

The second experimental design analysed the transcriptional response of *S*. *pneumoniae* R6 exposed to increasing concentrations of PEN (0.5X, 1X, 5X and 10X PEN MIC) for 15 minutes. These conditions were selected so the survival rate at the highest concentration would not be less than 30% to ensure the presence of metabolically active cells (Supplementary Fig. S1d). Of the 129 genes modulated, 42 were common to the time course design (underlined in Table 2). Among these, the expression of the *hcrA-grpE-dnaK-dnaJ* operon (spr0453–0456) coding for heat shock proteins and molecular chaperones, as well as the expression of the chaperone *groEL* (spr1722) and of a PTS system (spr0562–0563), was similarly increased in both designs. Specific to this incremental dosing design was the upregulation of operons involved in gluconate metabolism (spr0287-spr0290) and iron transport (spr1684-spr1687) (Table 2), the latter being of interest given the reported role of iron in PEN lethality^13,17^. Remarkably, the glutamine metabolic and transporter genes *glnR*, *glnA, glnP* and *glnQ* were again among the most downregulated genes (Table 2). To further validate this finding, and because *S*. *pneumoniae* isolates are highly polymorphic^27^, the time-course RNA-seq based transcriptomics were replicated with two PEN-susceptible clinical isolates (CCRI-21487 and CCRI-8970). Strikingly, while several genes had their expression changed in the clinical strains (Supplementary Tables S1 and S2), *glnR*, *glnA* and *glnQ* were among the few genes modulated in a common fashion in the presence of PEN in every condition tested (Table 3). Quantitative RT-PCR was used to monitor the expression of these genes at T3 compared to T0 and confirmed that PEN enhances the downregulation of *glnA*, *glnR*, *glnP* and *glnQ* in the three *S*. *pneumoniae* strains (Fig. 1).

**Table 2:** Genes modulated by PEN in *S*. *pneumoniae* R6 in the dose-response transcriptomic design.

Entry no. in R6 genome database^a^	Gene Symbol	Gene description	Log_2_FC (*q*-Value ≤ 0.05)^b,c^				
			NO PEN^d^	0.5X MIC	1X MIC	5X MIC	10X MIC
**Downregulated**							
spr0020	—	hypothetical protein	—	—	—	−1.26	−2.58
spr0078	*rpsD*	30S ribosomal protein S4	—	—	—	—	−1.30
spr0123	—	MutT/nudix family protein	—	—	—	—	−1.33
spr0127	*orf51*	hypothetical protein	−1.00	—	—	−1.62	—
spr0128	—	hypothetical protein	—	—	—	−1.27	—
spr0187	*rpsJ*	30S ribosomal protein S10	—	—	—	—	−1.26
spr0210	*adk*	adenylate kinase	—	—	—	−1.03	−1.43
spr0211	*infA*	translation initiation factor IF−1	—	—	—	—	−1.71
spr0216	*rplQ*	50S ribosomal protein L17	—	—	—	—	−1.34
spr0327	*aliA*	oligopeptide ABC transporter substrate-binding protein	—	—	—	—	−1.30
spr0381	*fabG*	3-ketoacyl-ACP reductase	—	−1.19	—	—	—
spr0382	*fabF*	3-oxoacyl-ACP synthase	—	−1.01	—	—	—
spr0383	*accB*	acetyl-CoA carboxylase biotin carboxyl carrier protein subunit	—	−1.16	—	—	—
spr0384	*fabZ*	(3R)-hydroxymyristoyl-ACP dehydratase	—	−1.05	−1.26	—	—
spr0386	*accD*	acetyl-CoA carboxylase subunit beta	—	−1.05	—	—	—
spr0388	—	hypothetical protein	—	−1.27	—	−1.30	−1.45
spr0398	*rpmB*	50S ribosomal protein L28	—	—	—	—	−1.87
**spr0443**	***glnR*** ^**e**^	**transcriptional repressor of the glutamine synthetase gene**	**—**	**—**	**—**	**−1.61**	**−2.38**
**spr0444**	***glnA*** ^**e**^	**glutamine synthetase, type I**	**—**	**—**	**—**	**−1.45**	**−2.06**
spr0682	*rpsP*	30S ribosomal protein S16	—	—	—	—	−1.26
spr0691	*bioY*	biotin synthase	—	—	—	—	−1.00
spr0714	*gph*	phosphoglycolate phosphatase	—	—	—	—	−1.02
spr0767	*IS1167*	transposase	—	—	—	—	−1.13
spr0861	*infC*	translation initiation factor IF-3	—	—	—	—	−1.20
spr0864	*lguL*	lactoylglutathione lyase	—	—	—	—	−1.20
spr1012	*rplU*	50S ribosomal protein L21	—	—	−1.41	—	—
**spr1120**	***glnP*** ^**e**^	**amino acid ABC transporter substrate-binding protein**	**—**	**—**	**—**	**—**	**−1.36**
**spr1121**	***glnQ*** ^**e**^	**amino acid ABC transporter ATP-binding protein**	**—**	**—**	**—**	**−1.05**	**—**
spr1144	*smf*	DNA processing protein DprA	—	—	—	−1.58	—
spr1208	—	hypothetical protein	—	—	−1.00	—	—
spr1210	—	hypothetical protein	—	—	—	−1.58	—
spr1409	—	glutathione S-transferase YghU	—	—	—	−1.17	−1.44
spr1410	*pacL*	calcium transporter P-type ATPase	—	—	—	−1.08	−1.26
spr1440	—	ATP-dependent RNA helicase	—	—	—	−1.24	−1.52
spr1604	*aqpZ*	aquaporin	—	—	—	−1.11	−1.60
spr1623	—	hypothetical protein	—	—	—	−1.11	—
spr1624	—	hypothetical protein	—	—	—	—	−1.26
spr1626	—	hypothetical protein	—	—	—	−1.31	—
spr1859	—	hypothetical protein	−1.00	—	—	−1.00	—
spr1883	—	hypothetical protein	—	—	−1.00	—	—
spr1912	—	hypothetical protein	—	—	−1.06	—	—
**Upregulated**							
spr0079	—	degenerative transposase	—	—	1.29	—	—
spr0096	—	LysM domain-containing protein	—	—	—	1.04	—
spr0102	*argG*	argininosuccinate synthase	—	1.00	—	—	—
spr0104	—	hypothetical protein	—	1.00	—	1.32	1.81
spr0151	—	hypothetical protein	—	—	—	1.05	—
spr0225	—	hypothetical protein	—	1.00	—	2.00	—
spr0247	*pulA*	alkaline amylopullulanase	—	—	—	—	1.84
spr0280	*celC*	PTS system cellobiose transporter subunit IIA	1.00	1.00	—	2.00	2.32
spr0282	*celD*	PTS system cellobiose transporter subunit IIC	—	1.00	—	—	—
spr0287	*kdgA*	keto-deoxy-phosphogluconate aldolase	—	1.00	1.32	—	—
spr0288	*kdgK*	2-keto-3-deoxygluconate kinase	1.00	1.00	—	—	1.58
spr0289	—	hypothetical protein	1.00	1.58	—	1.58	2.00
spr0290	*gno*	gluconate 5-dehydrogenase	1.00	1.00	—	—	—
spr0295	*PTS-EII*	PTS system transporter subunit IID	—	—	—	1.00	1.00
spr0311	—	hypothetical protein	—	—	—	1.38	—
spr0344	*rr03*	DNA-binding response regulator	—	—	—	—	1.36
spr0373	—	hypothetical protein	—	—	1.33	—	—
spr0453	*hrcA*	heat-inducible transcription repressor	—	1.32	—	1.72	1.83
spr0454	*grpE*	heat shock protein GrpE	—	1.03	1.11	1.70	1.79
spr0455	*dnaK*	molecular chaperone DnaK	—	1.30	1.23	1.88	2.18
spr0456	*dnaJ*	molecular chaperone DnaJ	—	1.23	1.74	—	1.85
spr0506	*bglH*	6-phospho-beta-glucosidase	—	—	—	—	1.24
spr0534	*glnH*	amino acid ABC transporter amino acid-binding protein	—	—	—	1.44	1.65
spr0562	*PTS-EII*	PTS system transporter subunit IIA	1.13	1.23	—	—	—
spr0563	—	hypothetical protein	—	1.03	—	—	—
spr0565	*bgaA*	beta-galactosidase	—	—	—	—	1.11
spr0613	*pyrF*	orotidine 5′-phosphate decarboxylase	1.09	1.14	—	1.27	—
spr0614	*pyrE*	orotate phosphoribosyltransferase	1,00	1.11	1.21	1.28	—
spr0615	—	hypothetical protein	1.25	1.17	—	—	—
spr0634	*tenA*	extracellular enzyme gene transcriptional regulator	—	1.00	—	—	—
spr0644	*Transposase_C*	transposase	1.00	1.00	—	—	—
spr0645	—	hypothetical protein	—	1.00	—	—	—
spr0664	—	acetoin utilization protein AcuB	—	—	1.12	1.12	—
spr0782	—	hypothetical protein	—	—	—	1.92	2.07
spr0791	*hsdS*	type I restriction-modification system S subunit	—	—	—	1.15	1.32
spr0793	*argR*	arginine repressor ArgR	—	1.14	—	1.38	—
spr0810	—	hypothetical protein	—	—	—	1.51	—
spr0811	—	hypothetical protein	—	—	—	1.22	1.42
spr0812	*ABC-NBD*	ABC transporter ATP-binding protein	—	—	—	1.00	—
spr0840	—	hypothetical protein	—	1.00	—	—	—
spr0887	*gpmB*	phosphoglycerate mutase	—	—	1.21	—	—
spr0946	—	hydrolase	—	—	—	1.00	—
spr0959	—	hypothetical protein	—	—	—	1.58	2.00
spr0997	—	hypothetical protein	—	1.04	—	—	—
spr1028	*gapN*	glyceraldehyde-3-phosphate dehydrogenase	1.28	1.32	1.24	1.51	1.65
spr1069	*lacG*	6-phospho-beta-galactosidase	—	—	—	1.00	1.50
spr1073	*lacD*	tagatose 1,6-diphosphate aldolase	—	1.00	1.05	1.41	1.96
spr1075	*lacB*	galactose-6-phosphate isomerase subunit LacB	—	1.20	—	—	1.93
spr1079	—	hypothetical protein	—	—	—	—	1.58
spr1080	—	hypothetical protein	—	—	—	—	1.58
spr1081	—	hypothetical protein	—	—	—	1.00	1.22
spr1097	*nirC*	formate/nitrate transporter	1.42	1.66	2.17	2.12	2.58
spr1112	—	hypothetical protein	—	—	1.21	—	—
spr1291	—	hypothetical protein	—	1.00	—	—	—
spr1293	*ABC-NBD*	ABC transporter ATP-binding protein	—	—	—	1.26	—
spr1382	*aliB*	peptide ABC transporter substrate-binding protein	1.06	1.06	1.13	1.67	1.86
spr1467	*rpsO*	30S ribosomal protein S15	—	—	1.11	—	—
spr1475	—	hypothetical protein	—	—	—	1.00	—
spr1527	*ABC-SBP*	sugar ABC transporter substrate-binding protein	—	—	—	—	1.32
spr1528	*PTS-EII*	PTS system transporter subunit IIBC	—	—	—	—	1.42
spr1536	*nanA*	neuraminidase A	—	1.58	—	2.00	2.58
spr1630	—	hypothetical protein	—	—	—	1.08	—
spr1646	—	hypothetical protein	1.58	1.00	—	1.58	—
spr1649	—	phosphate transporter PhoU	1.00	1.00	—	1.58	—
spr1667	*galT*	galactose-1-phosphate uridylyltransferase	—	—	1.32	1.00	1.50
spr1668	*galK*	galactokinase	—	—	—	1.12	1.42
spr1684	*fatD*	iron-compound ABC transporter permease	—	—	1.78	—	—
spr1685	*fatC*	iron-compound ABC transporter permease	—	1.00	—	—	—
spr1686	*fecE*	iron-compound ABC transporter ATP-binding protein	—	1.22	—	—	—
spr1687	*fatB*	iron-compound ABC transporter substrate-binding protein	—	1.12	—	—	—
spr1700	*treR*	trehalose operon transcriptional repressor	—	—	—	1.43	1.38
spr1715	*birA*	biotin–protein ligase	—	—	—	1.00	—
spr1721	—	transposase	—	1.00	—	—	—
spr1722	*groEL*	molecular chaperone GroEL	—	—	1.30	1.43	1.58
spr1800	—	hypothetical protein	1.00	—	—	1.22	1.22
spr1801	*ABC-NBD*	ABC transporter ATP-binding protein	—	—	1.22	—	—
spr1810	—	hypothetical protein	—	—	—	1.22	—
spr1837	*adhE*	bifunctional acetaldehyde-CoA/alcohol dehydrogenase	2.20	2.27	2.15	3.07	3.09
spr1866	*adh*	zinc-containing alcohol dehydrogenase	—	—	—	1.23	1.45
spr1895	*pstS*	phosphate ABC transporter substrate + B766-binding protein	—	—	—	1.25	1.43
spr1896	*pstC*	phosphate ABC transporter permease	—	—	1.38	—	—
spr1915	—	hypothetical protein	—	—	—	1.05	—
spr1953	—	hypothetical protein	—	1.00	—	—	—
spr1962	—	hypothetical protein	—	—	—	1.35	1.80
spr1983	—	hypothetical protein	—	1.00	—	1.93	1.93
spr2011	—	ribosomal subunit interface protein	—	—	—	1.41	1.57
spr2016	*transposase_G*	hypothetical protein	—	1.00	—	—	—

^a^Genes underlined are common between Table 1 and Table 2.

^**b**^Penicillin (PEN) was added at 0.5, 1, 5 and 10X MIC at T0 and the differential gene expression was tested between 15 min and T0. Genes included showed significant variations of log_2_ FC ≤ −1 or log_2_ FC ≥ +1 with a *q*-value ≤0.05.

^**c**^‘−’ means no significant change in expression.

^**d**^‘No PEN’ corresponds to the genes modulated in the untreated R6.

^**e**^Genes involved in glutamine metabolism are shown in bold.

**Table 3:** Genes modulated in a common fashion by PEN in *S*. *pneumoniae* R6, CCRI-8970 and CCRI-21487.

Entry no. in R6 genome database	Gene Symbol	Gene description	Condition	Log_2_FC (*q*-Value ≤ 0.05)^a,b^								
				R6			CCRI-8970			CCRI-21487		
				T1/T0	T2/T0	T3/T0	T1/T0	T2/T0	T3/T0	T1/T0	T2/T0	T3/T0
**Downregulated**												
**spr0443**	***glnR*** ^**e**^	**transcriptional repressor of the glutamine synthetase gene**	**NO PEN** ^**c**^	**—**	**—**	**—**	**—**	**−2.53**	**−2.35**	**—**	**—**	**—**
			**PEN 1X MIC** ^**d**^	**—**	**−1.79**	**−3.37**	**−2.08**	**−3.02**	**−2.93**	**—**	**−1.08**	**−2.32**
**spr0444**	***glnA*** ^**e**^	**glutamine synthetase, type I**	**NO PEN**	**—**	**—**	**—**	**−1.42**	**—**	**—**	**—**	**—**	**−1.26**
			**PEN 1X MIC**	**—**	**−1.62**	**−3.02**	**−1.75**	**−1.45**	**−1.65**	**—**	**−1.45**	**−2.82**
**spr1121**	***glnQ*** ^**e**^	**amino acid ABC transporter ATP-binding protein**	**NO PEN**	**—**	**—**	**—**	**−1.02**	**−1.55**	**−1.80**	**—**	**—**	**—**
			**PEN 1X MIC**	**—**	**−1.14**	**−1.83**	**−2.05**	**−2.63**	**−2.96**	**—**	**−1.10**	**−2.23**
**Upregulated**												
spr0307	*clpL*	ATP-dependent protease ATP-binding subunit	NO PEN	—	—	—	—	1.33	1.81	—	—	—
			PEN 1X MIC	—	—	2.16	1.30	2.03	2.22	—	—	1.13
spr0455	*dnaK*	molecular chaperone DnaK	NO PEN	1.34	—	1.08	—	1.50	1.49	—	—	—
			PEN 1X MIC	1.80	2.24	2.07	1.33	1.71	1.75	—	—	1.01
spr0456	*dnaJ*	molecular chaperone DnaJ	NO PEN	1.40	1.00	1.03	—	2.01	1.86	—	—	—
			PEN 1X MIC	1.57	1.91	1.73	1.80	1.97	2.10	—	—	1.15
spr1866	*adh*	zinc-containing alcohol dehydrogenase	NO PEN	—	—	1.03	—	—	—	—	1.11	—
			PEN 1X MIC	1.06	—	—	—	—	1.04	—	1.13	1.25
spr2011	—	ribosomal subunit interface protein	NO PEN	—	—	—	—	—	—	—	1.33	1.27
			PEN 1X MIC	—	1.40	1.94	—	—	1.06	1.31	1.55	2.15

^a^Penicillin (PEN) at 1 X MIC was added at T0 and the differential gene expression was tested for three time points (T1, T2 and T3). Genes included showed significant variations of log_2_ FC ≤ −1 or log_2_ FC ≥ +1 with a *q*-value ≤ 0.05.

^b^‘−’ means no significant change in expression.

^c^‘No PEN’ corresponds to the genes modulated in the untreated *S*. *pneumoniae*.

^**d**^‘PEN 1 X MIC’ corresponds to the genes modulated in the treated bacteria.

^**e**^Genes involved in glutamine metabolism are shown in bold.

### Modulation in glutamine levels in penicillin treated *S*. *pneumoniae*

**Figure 2:**
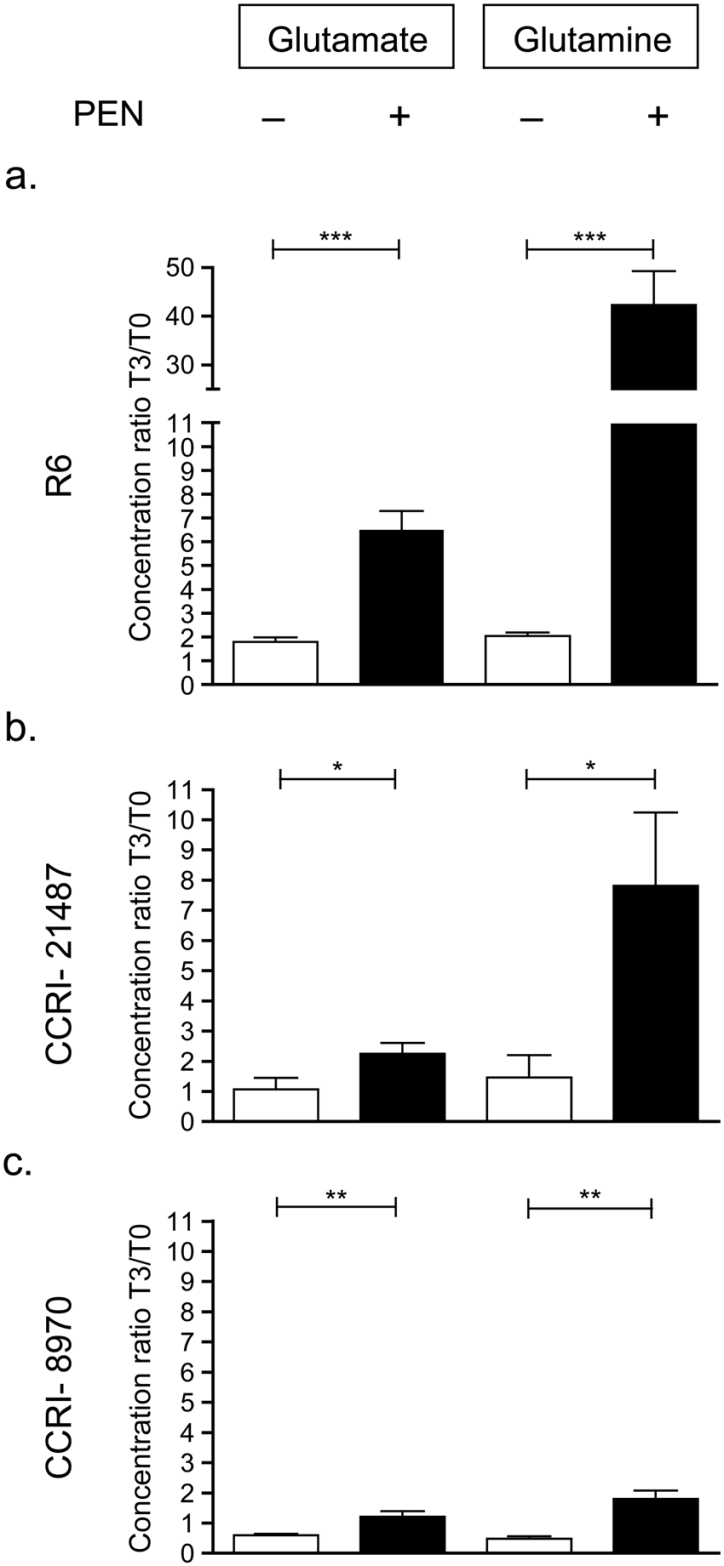
Quantification of intracellular levels of glutamine and glutamate by LC-MS. Glutamine and glutamate relative concentrations at time point T3 (corresponding to 40 min for R6, 28 min for CCRI-21487 and 85 min for CCRI-8970) over T0 in untreated (white bars) and PEN-treated (black bars) *S*. *pneumoniae* R6 (**a**), CCRI-21487 (**b**) and CCRI-8970 (**c**). The data was normalized according to the bacterial counts. Results are displayed as mean ± SD of three biological replicates and significant differences were determined by unpaired student t-test (*p ≤ 0.05, **p ≤ 0.01, ***p ≤ 0.001).

The transcriptomics data revealed a possible alteration to the metabolism of glutamine in *S*. *pneumoniae* upon exposure to PEN and this was further analysed using liquid chromatography coupled to mass spectrometry (LC-MS). The levels of glutamine and glutamate were quantified in the three *S*. *pneumoniae* R6, CCRI-8970 and CCRI-21487 strains in the presence or absence of PEN at time point equivalent to T3 above, in addition to the T0 baseline (Table 3). In *S*. *pneumoniae* R6 the level of glutamine increased close to 40-fold after exposure to PEN, whereas it increased just by 2 fold in untreated bacteria (Fig. 2a). The levels of glutamate changed more modestly, an increase of 6-fold, in bacteria treated with PEN (Fig. 2a). PEN also increased glutamine concentration in *S*. *pneumoniae* CCRI-21487 close to 8 fold (Fig. 2b), while glutamate level were only modestly increased (Fig. 2b). Glutamine and glutamate levels remained unchanged in untreated CCRI-21487 (Fig. 2b). As for *S*. *pneumoniae* CCRI-8970, glutamine level increased close to 2 fold after exposure to PEN, whereas it decreased by 0.5 fold in the untreated bacteria (Fig. 2c). Glutamate level remained unchanged after exposure to PEN and similarly to the level of glutamine, it decreased by around 0.5 fold in untreated CCRI-8970 (Fig. 2c).

### Glutamine confers low-level protection against penicillin in *S*. *pneumoniae* R6

**Figure 3:**
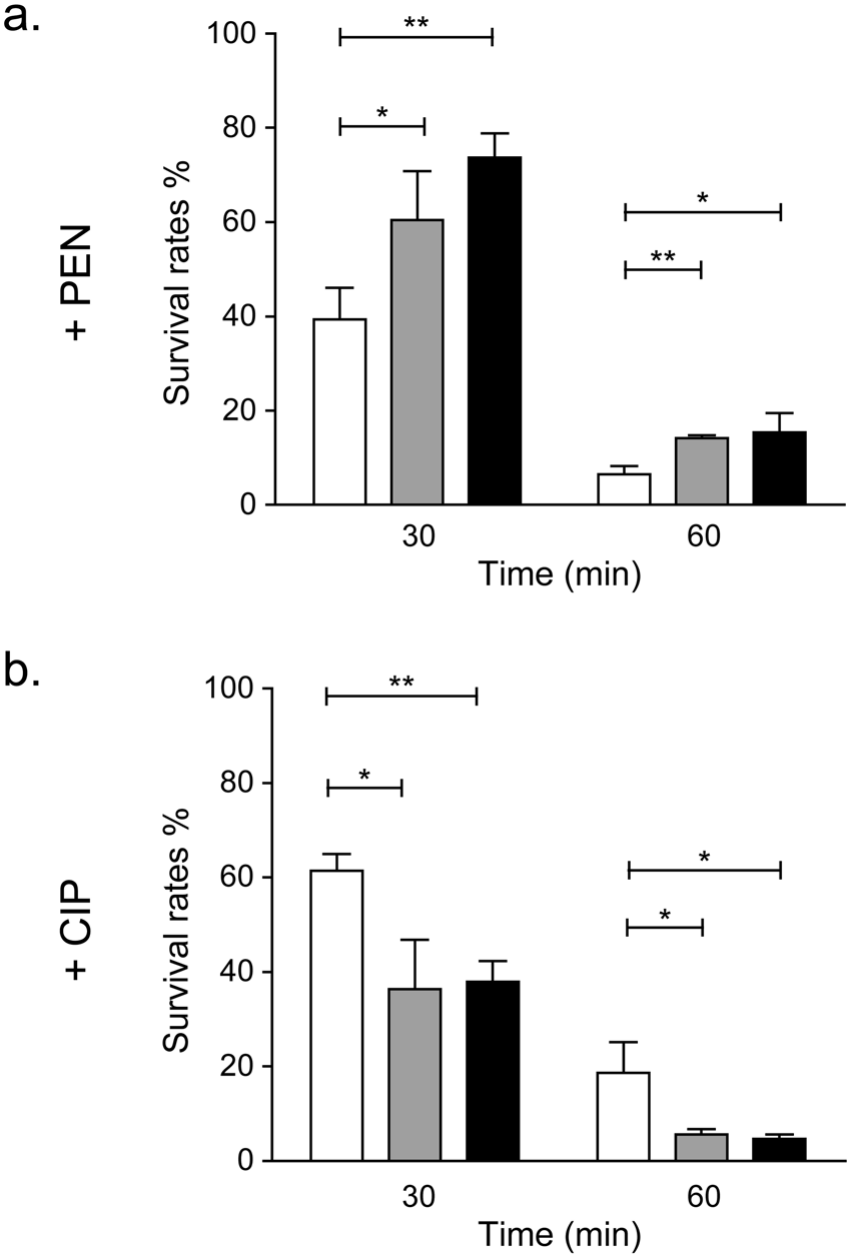
Protective role of glutamine against penicillin in *S*. *pneumoniae* R6. Survival rates at 30 min and 60 min of *S*. *pneumoniae* R6 in BHI media supplemented with penicillin (**a**) or ciprofloxacin (**b**) with the addition of 0 mM (white bars), 6 mM (gray bars) or 12 mM (black bars) glutamine. Untreated *S*. *pneumoniae* R6 was used as control. Results display the mean ± SD of at least three biological repeats and significant differences were determined by unpaired Student’s t test (*p ≤ 0.05, **p ≤ 0.01, ***p ≤ 0.001).

Penicillin increases glutamine levels (Fig. 2). We tested whether glutamine could protect against penicillin action. *S*. *pneumoniae* R6 was grown in BHI medium supplemented with glutamine at 0, 6 and 12 mM at early log-phase, half an hour before the addition of PEN at 1X MIC (Supplementary Fig. S2a). The survival rates at 30 min and 60 min were derived from the ratio of bacterial counts in the presence of PEN compared to untreated bacteria at each time point. Interestingly, glutamine at 6 mM and 12 mM conferred considerable protection against PEN by increasing survival rates at 30 min from 40% to 60% and from 40% to 75%, respectively (Fig. 3a). Survival rates also increased from 5% to 11% at 60 min in the presence of glutamine (Fig. 3a). The protection conferred by glutamine supplementation was specific to PEN and was not observed with ciprofloxacin (CIP), a fluoroquinolone antibiotic inhibiting DNA replication (Fig. 3b & Supplementary Fig. S2b). In fact in the case of CIP, glutamine at 6 mM or 12 mM was detrimental and apparently enhanced its lethality by decreasing survival rates from 60% to 38% and from 20% to 5% at 30 and 60 min, respectively (Fig. 3b).

### Inhibiting the glutamine synthetase GlnA renders *S*. *pneumoniae* more susceptible to penicillin

Because glutamine supplementation decreased susceptibility to PEN, we next sought whether inhibiting the glutamine synthetase GlnA would enhance PEN lethality. The MIC of PEN against *S*. *pneumoniae* R6 was determined in the presence of L-methionine sulfoximine (MSO), a specific inhibitor of GlnA^28–30^. Attempts to determine the toxicity of MSO in *S*. *pneumoniae* were not possible by microdilution and bacteria were growing albeit at a lesser density up to 32 mM by macrodilution. The larger culture volume of macrodilution (see Methods) facilitated measurements. Several concentrations of MSO were tested in combination with PEN, and 0.5 mM MSO was found to have maximal effect on PEN susceptibility while alone having minimal activity against *S*. *pneumoniae*. Indeed, 0.5 mM MSO increased the susceptibility of *S*. *pneumoniae* R6 to PEN by four-fold, from a MIC of 0.03 µg/mL in the absence of MSO to a MIC of 0.008 µg/mL (Table 4). Addition of glutamine at 12 mM completely reverted this PEN hyper-susceptibility phenotype (Table 4). A two- to four-fold sensitization to PEN induced by MSO was also observed in the PEN-susceptible *S*. *pneumoniae* clinical isolates CCRI-21487 and CCRI-8970, but also in PEN-resistant clinical isolates (CCRI-1397, CCRI-1414 and CCRI-1983) (Table 4). Again, glutamine fully (or partially in the case of CCRI-1983) rescued the hyper-susceptibility phenotype (Table 4).

**Table 4:** Inhibition of GlnA by L-Methionine Sulfoximine sensitizes *S*. *pneumoniae* to PEN.

Condition	MIC of PEN (µg/mL)^a,b^					
	R6	CCRI-8970	CCRI-21487	CCRI-1397	CCRI-1414	CCRI-1983
CAMHB^c^	0.03	0.015	0.03	1	2	8
CAMHB + MSO^d^	**0.008**	**0.008**	**0.015**	**0.5**	**1**	**2**
CAMHB + MSO + Gln^e^	0.03	0.015	0.03	1	2	4

^a^MIC of PEN: Minimum Inhibitory Concentration of penicillin.

^**b**^Average of three biological replicates.

^**c**^CAMHB: Cation Adjusted Muller Hinton Broth.

^**d**^MSO: L-Methionine Sulfoximine added at 0.5 mM.

^**e**^Gln: glutamine added at 12 mM.

## Discussion

Gene expression modulation is central to bacterial adaptation and can mirror the cellular response to stress-induced damages. In this study, we used RNA-seq to assess the metabolic consequences of exposure to PEN in *S*. *pneumoniae*. A number of genes previously shown to be implicated in PEN resistance were detected, including *ciaRH* ^19^, *pstS* ^19,31^ and *clpL* ^32,33^. The transcriptional regulator *CiaR* was previously shown to influence on natural competence and susceptibility to β-lactams in *S*. *pneumoniae* ^34^. It is known to activate fourteen promoters^35^ and many of the genes under its control had indeed increased expression in the presence of PEN, such as the foldase *prsA*, the acetyl xylan esterase *axe1*, the maltodextrin phosphorylase *malP*, the serine protease *htrA*, the chromosome segregation protein *spoOJ* and the hypothetical protein coding genes spr0782 and spr0931 (Table 1). CiaR also drives the expression of small regulatory non-coding RNAs^36^ and one target of such RNA (the formate/nitrate transporter *nirC*) was found to be overexpressed (Table 1). Apart from the CiaRH regulon, adenylate kinase, glutathione S-transferase, the fatty acid and phospholipid-related genes *fabK*, *fabG*, *fabF*, *fabZ* and *accD* and a gene from the MutT/nudix family whose expression was decreased in our RNA-seq data had previously been shown to be downregulated by PEN using microarrays^19^, so is the case for the overexpression of LysM domain-containing proteins and molecular chaperones (Tables 1 & 2).

Common to all *S*. *pneumoniae* strains and transcriptomics designs was the downregulation of the *glnRA* and *glnPQ* operons (Table 3). The transcriptional regulator *glnR* mediates the repression of its own *glnRA* operon, the *glnPQ-zwf* operon and the *gdhA* gene by binding to a conserved operator sequence^37^. The gene *glnA* encodes glutamine synthetase which is responsible for the conversion of glutamate and ammonium into glutamine^37^. GlnA also indirectly influences the transcription of its own operon by stimulating the binding of GlnR. The *glnPQ* genes are coding for a transporter involved in glutamine scavenging^37^. Targeted metabolomics using LC-MS revealed an increase in glutamine concentrations following exposure to PEN in all three *S*. *pneumoniae* strains studied (Fig. 2). The increase is considerable with 4-to-20 fold increase compared to the untreated cells control. Since high concentration of glutamine down-regulates the *glnRA* and *glnPQ* operons^37,38^, the decreased expression of those two operons (Table 3) are most likely due to the penicillin-induced increase in glutamine levels (Fig. 2). Glutamate levels increased moderately (2–4 fold) in the *S*. *pneumoniae* R6, CCRI-21487 and CCRI-8970 when compared to untreated cells at the same time point (Fig. 2). Intriguingly, glutamine levels were increased two-fold in R6 during growth (Fig. 2a) but not in the two other strains. One possible candidate is *glnH* (spr0534) coding for an ABC transporter that binds glutamine and glutamate^39^. Indeed, *glnH* remained constant in R6 untreated cells but downregulated in untreated CCRI-8970 and CCRI-21487 (Tables S1 and S2). These RNA seq data were confirmed by qRT-PCR (Supplementary Fig. S3). Upon penicillin treatment, *glnH* is downregulated in the two clinical isolates but upregulated in R6 (Supplementary Fig. S3). This differential expression of *glnH* in R6 may contribute to the higher uptake and accumulation of glutamate and glutamine observed in R6 (Fig. 2).

The mechanism by which PEN increases the levels of glutamine is still unclear. However, glutamine is a major nitrogen donor for the synthesis of cell building blocks and is used by the aminotransferase GlmS to convert fructose-6-phosphate into glucosamine-6-phosphate (Glc-6P)^40^. GlmS occupies a central position between glycolysis and peptidoglycan synthesis in catalysing the first of a series of reactions leading to the cell wall precursor UDP-N-acetylglucosamine^40^. Inactivation or chemical inhibition of GlmS was shown to synergize with a broad set of cell wall synthesis inhibitors in *Staphylococcus aureus* ^41,42^. Similarly, disruption of the downstream phosphoglucosamine mutase GlmM increased the susceptibility of *Streptococcus gordonii* to PEN^43^ in addition to decrease methicillin resistance in *S*. *aureus* without affecting the production of endogenous PBPs^44–46^. Similarly, inhibiting the wall teichoic acid transporter protein TarG was shown to potentiate the activity of imipenem against methicillin-resistant *Staphylococcus aureus* ^47^. Consistent with these findings, here we observed that the inhibition of *GlnA* leads to penicillin sensitization in *S*. *pneumoniae* clinical isolates (Table 4). Chemical inhibition of GlmS in methicillin-resistant *S*. *aureus* ^42^ and of GlnA in PEN-resistant *S*. *pneumoniae* clinical isolates (Table 4) decreased their levels of resistance. Conversely, the mere addition of glutamine was found to be partly protective against the action of penicillin (Fig. 3). Glutamine is also a co-factor for the amidation of the second amino acid residue of lipid II by MurT/GatD, which is required for efficient cross-linking of the peptidoglycan^48^. It remains to be established whether glutamine accumulation is a secondary response from bacteria to PEN attack against the peptidoglycan assembly machinery or if the role of PEN is more direct, for example by hampering the use of glutamine by GlmS or by MurT/GatD. Additional work may shed further light on this.

In an era of ever increasing antibiotic resistance and shortage of new molecules, new strategies are required for increasing the activity of current antibiotics against sensitive and resistant bacteria. Our study showed a new link between glutamine metabolism and PEN susceptibility. The search for antibiotic adjuvants is now an intensive field of investigations^8^. Metabolites are now emerging as possible adjuvants and one possible strategy is the inhibition of metabolic pathways. For example exogenous alanine was shown to revert kanamycin resistance in a number of bacterial species^49^ and tetracycline resistance and thiamine biosynthesis were linked in *S*. *pneumoniae* ^25^ while bacterial resistance to tetracycline can be reversed using tryptophan analogues^50^. Further investigations are warranted in the attempt of restoring PEN activity using strategies to lower cellular glutamine levels. For example, untargeted metabolomics experiments may help elucidating the mechanism by which PEN leads to cell death by revealing the extent of metabolic alterations associated with it. This may have added benefits as glutamine was also shown to be involved in virulence, and deletion of *glnA* and *glnP* led to attenuated colonization^38^ and to decreased bloodstream invasiveness and survival in the lungs^38^, respectively. While still speculative, interfering with the metabolism of glutamine such as inhibiting GlnA would increase PEN susceptibility but also reduces virulence and drug-like leads against this target may thus serve dual purposes when paired with existing β-lactam antibiotics.

## Methods

### Bacterial strains and growth conditions

*S*. *pneumoniae* strains were grown in brain heart infusion broth (BHI, Becton Dickinson), or in blood agar containing 5% defibrinated sheep’s blood (Becton Dickinson). Cultures were incubated for 16 to 24 hours in a 5% CO_2_ atmosphere at 35 ^°^C as previously described^51^. All strains were maintained frozen at −80 ^°^C in BHI containing 15% glycerol. For the RNA-seq experiments, *S*. *pneumoniae* was grown in BHI broth at 35 °C until early log-phase (OD_600_ = 0.11). At this time (T0), cultures were divided into 18 tubes for the time-course design, half of which contained 1 X MIC PEN and incubated until survival rates of 95–75% (T1), 75–60% (T2) and 60–40% (T3) (6 tubes each). These time points correspond to 15, 30 and 40 min for R6, 8, 18 and 28 min for CCRI-21487 and 45, 65 and 85 min for CCRI-8970. For the dose-response design, at T0 *S*. *pneumoniae* R6 culture was divided into 30 tubes, half of which contained either 0.5X, 1X, 5X or 10X PEN MIC, and incubated for 15 min. At each time point, a serial dilution in PBS 1x was carried out to determine survival rates.

### Antibacterial susceptibility testing

MIC of PEN or CIP were determined with E-test strips (AB BioMérieux) on Müller-Hinton agar plates supplemented with 5% sheep blood (Becton Dickinson) using manufacturer’s instructions. The MICs were further confirmed by the microdilution method in a 96 wells plate according to the Clinical Laboratory Standards Institute (CLSI) guidelines in 0.1 ml Cation Adjusted Müller-Hinton Broth (CAMHB) supplemented with 5% lysed sheep blood (Difco). All MIC were determined from three independent biological replicates, each replicate being further assessed in technical duplicates. PEN and CIP were purchased from Sigma-Aldrich.

Macrodilution was performed in triplicate in a volume of 3 ml in CAMHB or CAMHB supplemented with 12 mM glutamine. *S*. *pneumoniae* inoculum were prepared by suspending colonies grown overnight on TSA agar (Becton Dickinson) using 1× PBS to achieve a turbidity of 0.5 McFarland (1 × 10^8^ CFU/ml). Fifteen microliters of this suspension was inoculated to the CAMBH broth to reach a concentration of 5 × 10^5^ CFU/mL. MICs of PEN were determined in the presence or absence of varying concentrations (0.008 to 32 mM) of L-methionine sulfoximine (MSO; Sigma-Aldrich) but 0.5 mM was the lowest MSO concentration to allow maximal effects on the MIC of PEN. For sensitive strains, the range of PEN concentrations tested varied between 0.004 and 0.12 µg/mL in doubling dilutions. For resistant isolates this varied from 0.06 to 8 µg/mL. Tubes were incubated at 35 ^°^C and turbidity was checked after 20–24 h.

### RNA sequencing

Total RNA was isolated at different time points from *S*. *pneumoniae* R6, CCRI-8970 and CCRI-21487 grown in BHI using the RNeasy Mini Kit (Qiagen) according to manufacturer’s instructions. The RNAs were treated with DNase I (Ambion) to avoid any DNA contamination. RNAs were quantified using 2100 BioAnalyzer RNA6000 Nano chips (Agilent) and 1 µg of total RNA was treated with Ribo-Zero^TM^ Magnetic Kit for Gram-Positive Bacteria (Epicentre). The rRNA-depleted samples were purified using RNeasy MinElute Cleanup kit (Qiagen). RNA-seq libraries were produced from 50 ng of rRNA-depleted samples using the ScriptSeq^TM^ v2 RNA-Seq Library Preparation Kit (Epicentre). The libraries were analysed using 2100 BioAnalyser High Sensitivity DNA Chips (Agilent) and quantified by QuantiFluor. The libraries were pooled, diluted to 8 pM and sequenced on an Illumina HiSeq system using a 101 bp paired-ends reads protocol.

### RNA-seq data analysis

The *S*. *pneumoniae* R6 genome^52^ (NCBI accession number AE007317) was used as reference. Sequencing reads were aligned to the *S*. *pneumoniae* R6 genome and analysed using the software Rockhopper^53^ with default settings. The RNA-seq data are available under the accessions number from SAMN07298959 to SAMN07298986 under the BioProject PRJNA392406.

### Quantitative reverse transcription (qRT-PCR)

The qRT-PCR experiment was done as previously described^25^. Briefly, total RNAs were extracted as described above and treated with DNase I (Ambion) to avoid any DNA contamination. RNA quality and integrity was assessed by agarose gel electrophoresis. cDNAs were generated from 50 ng of total RNA using the Superscript II reverse transcriptase (Invitrogen) and random hexamers according to the manufacturer’s instructions. qRT-PCR assays were carried out on a Bio-Rad Cycler using SYBR Green I. A final volume of 10 µl was used for each reaction containing specific primers (Table S4) and iQ SYBR Green Supermix (Bio-Rad). The relative quantitation of gene expression was performed using the relative standard curve method. All qRT-PCR data were normalized according to the amplification signals of 16S rRNA.

### Metabolites extraction

*S*. *pneumoniae* strains were grown in BHI broth at 35 ^°^C until they reached early log-phase (OD_600_ = 0.11). At this time (T0), cultures were divided into 6 tubes of 10 mL, half of which contained 1X MIC PEN, and incubated for a duration equivalent to time point T3 of the time-course RNA-seq experiments. Samples were rapidly quenched by submersion into dry ice/ethanol for 20 sec. The quenched samples were centrifuged at 1500 RCF for 10 min at 4 ^°^C then washed twice with ice-cold PBS 1× and centrifuged for 5 min at 4 ^°^C. Pellets were flash frozen in liquid nitrogen before being stored at −80 ^°^C until use. Cell lysis and protein denaturation was achieved by addition of 200 µL of cooled methanol: water (4:1) supplemented with a mixture of deuterated glutamine and glutamate standards (at 300 and 500 ng/mL respectively) along with 100 mg acid washed glass beads (≤106 µm, Sigma) to the pellets followed by agitation in a bead beater at 4 ^°^C for 6 cycles of 1 min agitation and 1 min cooling (Mini-BeadBeater-24, BioSpec Products, Inc.). Samples were then centrifuged at 13000 rpm for 5 min at 4 ^°^C. Supernatant (150 µL) were speed-vac dried for 65 min at medium heat. Lyophilised samples were kept at −80 ^°^C until further analysis. The LC-MS data were normalized according to the bacterial count obtained by plating on Trypticase soy agar supplemented with 5% sheep blood (BD).

### LC-MS

Lyophilised samples were resuspended in 150 µL of 50:50 of mobile phase A: mobile phase B (MPA: MPB), sonicated 5 min and filtrated on a 0.45 µm filter before injection. Liquid chromatography was performed on an Acquity UPLC I-Class binary pump system. The MPA consisted of 0.1% formic acid in water and the MPB consisted of 0.1% formic acid in acetonitrile. The column used was a BEH Amide 1.7 µM × 2.1 mm × 150 mm (Waters Corporation, part no. 186004802) at 45 ^°^C. A VanGuard pre-column with 2.1 × 5 mm filter unit (Waters corporation, part no 205000343) was used to protect the analytical column from impurities. Gradient conditions were: 0.01 to 0.1 min = 99% B; 0.1 to 7.00 min = 99 to 50% B; 7.00 to 7.10 min = 50% to 99% B; 7.10 to 10.00 min = 99% B. Injection volume was 2.0 µL, and flow rate was 0.4 mL/min. Single reaction monitoring (SRM) analysis were performed on a Q-TOF Synapt G2-*Si* system with an electrospray ionization source (ESI) in positive ionization mode. Source conditions and MS parameters were optimized by direct infusion of standards in sensitivity mode. MS parameters included: capillary voltage 2.75 kV, source temperature 120 ^°^C, desolvation temperature 325 ^°^C and desolvation gas 900 L*min^−1^. The specific SRM transitions for quantification were: glutamate 147.0764 > 130.0499, glutamine 148.0604 > 130.0499, deuterated glutamate 153.0979 > 135.0838 and deuterated glutamine 152.1155 > 135.0838. All data were collected by MassLynx software version 4.1. Deuterated glutamate (DLM-357-0.25) and deuterated glutamine (DLM-1826-0.1) were purchased from Cambridge Isotope Laboratories. Calibration curves of both metabolites using seven concentration points (23-46-94-187-375-750 and 1500 ng/mL) were obtained by comparison of the area ratios. Deuterated standards and QC samples at low (80 ng/mL), medium (320 ng/mL) and high (640 ng/mL) concentration were directly diluted in a pool of matrix from *S*. *pneumoniae* untreated samples. The relative concentrations of intracellular glutamine and glutamate in metabolite extracts were determined against the calibration curves. For a detailed description of the LC-MS analysis, see the supplementary data (Supplementary Tables S5 & S6).

### Statistical analysis

Statistical analysis for the qRT-PCR experiment was done using a split plot design and Fisher’s F-test using the SAS software. Significant differences in the LC-MS quantification of glutamine and glutamate and in survival rates in the presence of glutamine were determined using unpaired (two-tailed) Student’s t test in GraphPad Prism. Comparative data with a *p* value ≤ 0.05 were considered statistically different.

## Electronic supplementary material

Supplementary information
