# Supplementary material for: Penicillin induces alterations in glutamine metabolism in Streptococcus pneumoniae
Source: Sci Rep. 2017 Nov 6;7:14587. doi: 10.1038/s41598-017-15035-y (PMC5673960; doi:10.1038/s41598-017-15035-y)
Supplement: Supplementary file 1 — Supplementary information [file 41598_2017_15035_MOESM1_ESM.pdf]

Penicillin induces alterations in glutamine metabolism in *Streptococcus pneumoniae*.

Jessica El Khoury[1], Nancy Boucher[1], Michel G. Bergeron[1], Philippe Leprohon[1], Marc Ouellette[1*]

[1]Centre de Recherche en Infectiologie du Centre de Recherche du CHU de Québec and Département de Microbiologie, Infectiologie et Immunologie, Faculté de Médecine, Université Laval, Québec, Québec, Canada

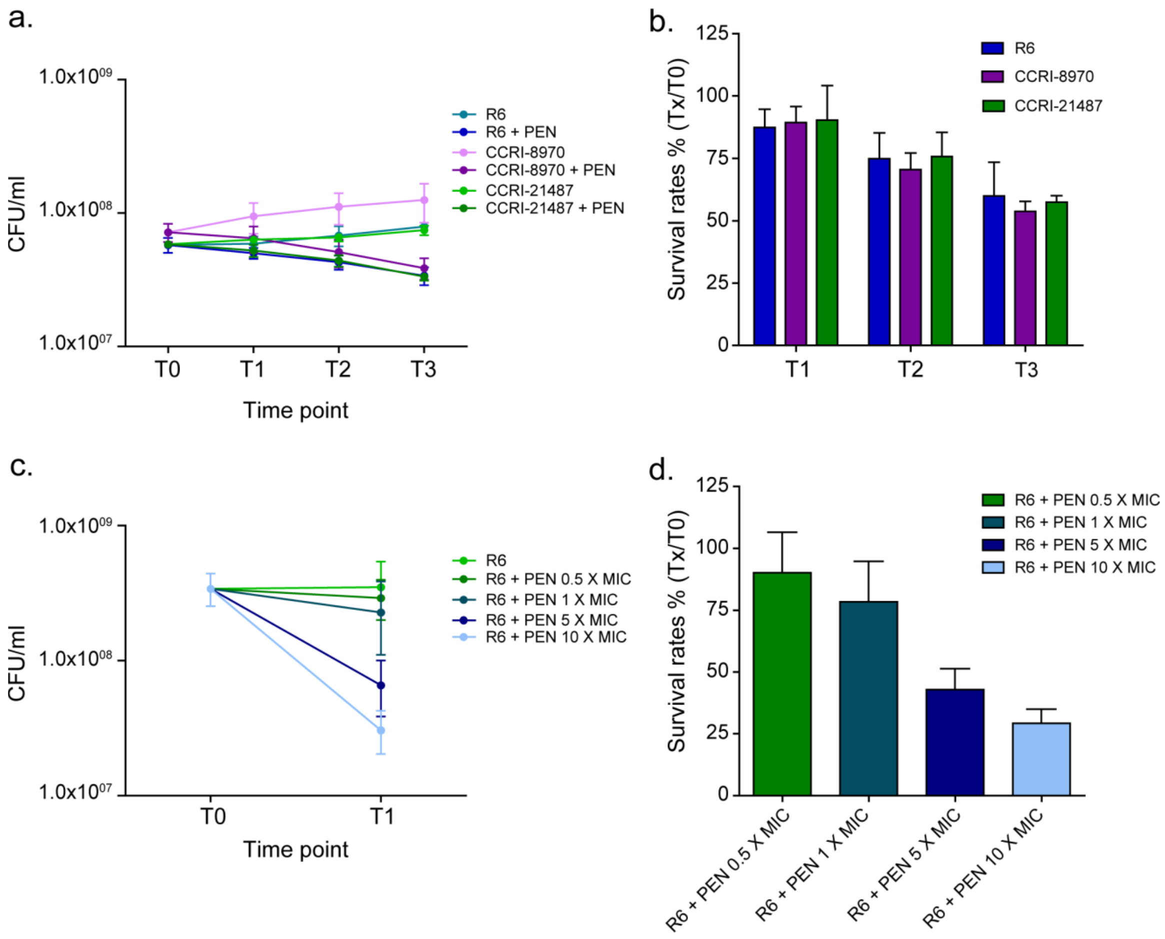

Supplementary Fig. S1: Growth curves of *S. pneumoniae* R6, CCRI-8970 and CCRI-21487 in BHI media alone or with the addition of penicillin (PEN) at 1X MIC for the time-course transcriptomic design (a). Survival rates at T1, T2 and T3 for the three strains (b). These time points correspond to 15, 30 and 40 min for R6, 8, 18 and 28 min for CCRI-21487 and 45, 65 and 85 min for CCRI-8970. Growth curves of *S. pneumoniae* R6 in BHI media alone or with the addition of PEN at 0.5X, 1X, 5X and 10X MIC (c) and the survival rates after 15 min (T1) for each concentration (d).

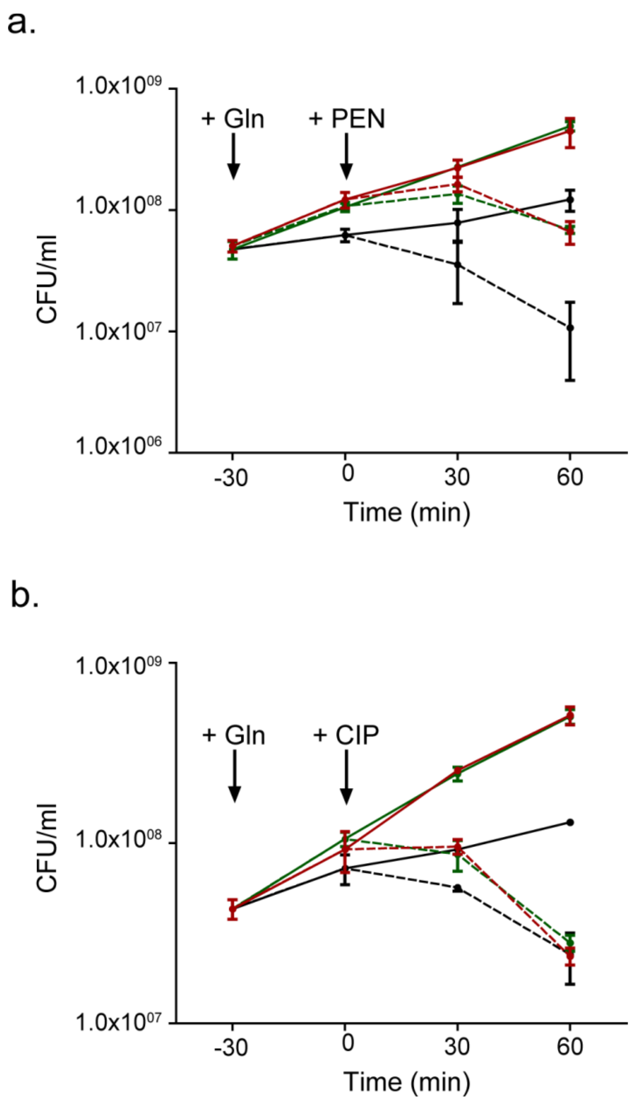

Supplementary Fig. S2: Growth curves of *S. pneumoniae* R6 in BHI media alone (black thin line) or supplemented with glutamine at 6 mM (green thin line) or 12 mM (red thin line) and of *S. pneumoniae* R6 in BHI media supplemented with PEN (at 1X MIC) (a) or CIP (at 2X MIC) (b) alone (black dashed line) or along with glutamine at 6mM (green dashed lines) or 12mM (red dashed lines). Glutamine was added half an hour (-30) before adding the antibiotic (0).

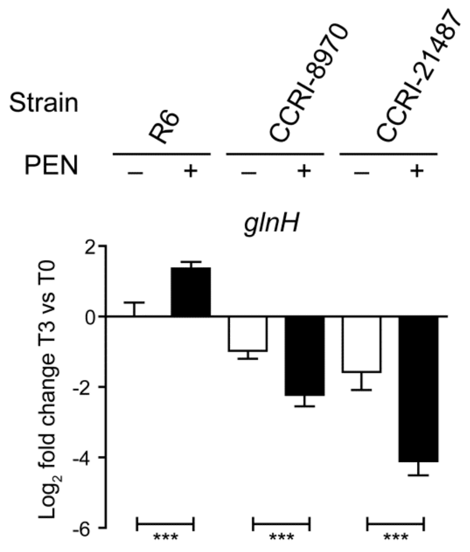

Supplementary Fig. S3: Penicillin-induced modulation of *S. pneumoniae glnH* expression by qRT-PCR.

Expression of *glnH* was validated by qRT-PCR. RNA levels were normalized based on the amplification signals of 16S ribosomal RNA. Graphs show the $\log_2$ fold change of expression at time T3 (corresponding to 40 min for R6, 28 min for CCRI-21487 and 85 min for CCRI-8970) over T0 in untreated (white bars) and PEN-treated (black bars) *S. pneumoniae* isolates. Results are displayed as mean ± SD of three biological replicates and significant differences are identified as determined by split plot design and Fisher's F-test (*** $p \leq 0.001$).

Supplementary Table S1: Genes modulated by PEN in *S. pneumoniae* CCRI-8970 in the time-course transcriptomic design.

			Log$_2$FC (q- Value ≤ 0.05)					
CCRI-8970			No PEN			PEN at 1X MIC		
**Entry no. in R6 genome database**	**Gene**	**Protein**	**45 min**	**65 min**	**85 min**	**45 min**	**65 min**	**85 min**
**Downregulated**								
spr0014	*transposase_A*	transposase	-	-2.06	-1.89	-	-1.98	-2.06
spr0031	-	hypothetical protein	-	-	-	-2.00	-	-
spr0052a	-	hypothetical protein	-	-	-	-	-	-1.00
spr0087	-	hypothetical protein	-	-	-1.54	-	-1.71	-1.77
spr0127	*orf51*	hypothetical protein	-	-	-	-	-	-1.00
spr0128	-	hypothetical protein	-	-	-	-1.42	-	-
spr0192	*rpsS*	30S ribosomal protein S19	-	-	-1.69	-	-1.97	-2.11
spr0442	-	hypothetical protein	1.23	-2.10	-1.60	-	-1.28	-
spr0443	*glnR*	transcriptional repressor of the glutamine synthetase gene	-	-2.53	-2.35	-2.08	-3.02	-2.93
spr0444	*glnA*	glutamine synthetase, type I	-1.42	-	-	-1.75	-1.45	-1.65
spr0480	-	hypothetical protein	-	-2.27	-1.98	-1.16	-1.87	-1.90
spr0489	-	hypothetical protein	-	-	-	-1.07	-	-
spr0532	*glnP*	amino acid ABC transporter permease	-	-	-1.20	-1.92	-1.82	-1.82
spr0533	*glnP*	amino acid ABC transporter permease	-	-1.42	-1.75	-2.00	-2.30	-2.46
spr0534	*glnH*	amino acid ABC transporter amino acid-binding protein	-1.98	-	-1.44	-2.40	-2.48	-2.76
spr0602	*ABC-NBD*	sodium ABC transporter ATP-binding protein	-2.00	-2.00	-2.00	-	-2.00	-2.00
spr0709	-	hypothetical protein	-1.11	-2.46	-2.14	-1.58	-2.40	-2.38
spr0840	-	hypothetical protein	-	-	-3.32	-1.32	-3.32	-2.32
spr0863	*rplT*	50S ribosomal protein L20	-	-	-1.36	-	-1.75	-1.67
spr0866	*pyrD*	dihydroorotate dehydrogenase 1B	-	-	-	-	-	-1.28
spr0941	-	hypothetical protein	-	-	-	-	-	-1.78
spr0942	*ccrB*	hypothetical protein	-	-1.49	-	-1.15	-	-
spr1003	-	hypothetical protein	-	-1.48	-	-	-	-
spr1051	*acoA*	TPP-dependent acetoin dehydrogenase subunit alpha	-2.15	-	-	-1.05	-	-
spr1075	*lacB*	galactose-6-phosphate isomerase subunit LacB	5.60	-1.75	-1.58	-	-1.33	-
spr1120	*glnP*	amino acid ABC transporter substrate-binding protein	-	-	-	-1.30	-1.69	-2.04
spr1121	*glnQ*	amino acid ABC transporter ATP-binding protein	-1.02	-1.55	-1.80	-2.05	-2.63	-2.96
spr1183	-	ABC transporter ATP-binding protein	-	-	-	-1.07	-	-
spr1241	-	hypothetical protein	-	-1.56	-1.28	-	-1.19	-
spr1346	-	hypothetical protein	-	-	-	-1.00	-	-
spr1392	-	hypothetical protein	3.86	-	-	-	-1.49	-
spr1503	-	hypothetical protein	-	-	-	-	-	-1.40
spr1582	-	DNA-directed RNA polymerase subunit omega	-	-1.83	-1.44	-	-1.62	-1.83
spr1593	-	hypothetical protein	-	-	-	-	-	-1.35
spr1603a	-	hypothetical protein	-	-	-	-	-	-1.58
spr1691	*scpA*	segregation and condensation protein A	-	-1.87	-1.58	-1.16	-1.50	-1.53
spr1806	-	cell wall surface anchor family protein	-	-1.37	-	-1.25	-	-
spr1828	*transposase_A*	transposase	-	-2.27	-2.01	-	-2.09	-2.18
spr1874	*marR*	MarR family transcriptional regulator	-	-	-	-1.10	-	-
spr1933	*rgg*	positive transcriptional regulator of glucosyltransferase and Spp phenotype	-	-	-	-1.17	-	-
spr1963	*adh2*	iron-containing alcohol dehydrogenase	-	-1.58	-1.58	-1.58	-	-
spr1964	*fucI*	L-fucose isomerase	-	-	-	-1.58	-	-
spr1973	*fcsK*	fucose kinase	-	-	-1.00	-1.00	-	-1.00
**Upregulated**								
spr0060	*PTS-EIIB*	PTS system transporter subunit IIB	-	-	-	-	1.81	1.58
spr0136	*glycosyltransferase*	glycosyl transferase	-	-	-	-	-	1.58
spr0152	-	hypothetical protein	-	-	-	-	-	1.22
spr0173	*spxA*	transcriptional regulator Spx	-	-	-	-	-	1.02
spr0307	*clpL*	ATP-dependent protease ATP-binding subunit	-	1.33	1.81	1.30	2.03	2.22
spr0390	*nusB*	transcription antitermination protein NusB	-	-	-	-	-	1.12
spr0399	*asp23*	hypothetical protein	-	-	1.27	-	1.58	1.64
spr0455	*dnaK*	molecular chaperone DnaK	-	1.50	1.49	1.33	1.71	1.75
spr0456	*dnaJ*	molecular chaperone DnaJ	-	2.01	1.86	1.80	1.97	2.10
spr0458	-	hypothetical protein	-	-	-	-	1.55	1.58
spr0459	*ABC-NBD*	ABC transporter ATP-binding protein	-	1.57	1.53	1.35	1.67	1.62
spr0656	*clpP*	ATP-dependent Clp protease proteolytic subunit	-	-	-	-	1.26	1.49
spr0661	*livM*	branched chain amino acid ABC transporter permease	-	-	-	-	1.18	1.24
spr0664	-	acetoin utilization protein AcuB	-	-	-	-	-	1.28
spr0697	-	hypothetical protein	-	-	-	-	-	1.38
spr0698	-	hypothetical protein	-	-	-	-	1.30	1.58

Supplementary Table S1: Genes modulated by PEN in *S. pneumoniae* CCRI-8970 in the time-course transcriptomic design *(Continued)* .

			Log$_2$FC (*q*- Value ≤ 0.05)					
CCRI-8970			No PEN			PEN at 1X MIC		
**Entry no. in R6 genome database**	**Gene**	**Protein**	**45 min**	**65 min**	**85 min**	**45 min**	**65 min**	**85 min**
**Upregulated**								
spr0707	*ciaR*	DNA-binding response regulator CiaR	-	-	-	-	-	1.30
spr0708	*ciaH*	sensor histidine kinase CiaH	-	-	-	-	-	1.29
spr0726	-	hypothetical protein	-	1.83	1.46	-	1.36	-
spr0740	*rpsT*	30S ribosomal protein S20	-1.41	2.76	1.29	1.72	-	-
spr0777	-	hypothetical protein	-	-	-	-	-	1.03
spr1063	*ptsH*	phosphocarrier protein HPr	-	-	-	-	-	1.22
spr1064	*nrdH*	glutaredoxin-like protein	-	-	-	2.29	1.69	1.35
spr1092	*truB*	tRNA pseudouridine synthase B	-	-	-	-	1.32	-
spr1100	*ldh*	L-lactate dehydrogenase	-	-	-	1.01	-	1.18
spr1139	*cutC*	copper homeostasis protein CutC	-	-	-	-	-	1.06
spr1144	*smf*	DNA processing protein DprA	-	-	-	-	1.18	1.24
spr1172	*crcB*	camphor resistance protein CrcB	-	-	-	-	-	1.32
spr1221	-	hypothetical protein	-	-	-	-	1.00	1.17
spr1344	*glpF*	glycerol uptake facilitator protein	-	1.84	1.20	1.25	1.21	1.14
spr1376	*patB*	aminotransferase, class II	-	-	-	-	-	2.00
spr1382	*aliB*	peptide ABC transporter substrate-binding protein	-	-	-	-	-	1.03
spr1385	-	hypothetical protein	-1.77	1.74	2.33	1.52	2.21	1.70
spr1430	*dpr*	non-heme iron-containing ferritin	-	-	-	-	1.32	-
spr1435	*apt*	adenine phosphoribosyltransferase	-	1.65	1.30	-	1.33	1.27
spr1449	-	hypothetical protein	-	-	-	-	2.17	2.46
spr1551	-	hypothetical protein	-	-	-	-	1.38	-
spr1601	-	hypothetical protein	-	-	-	-	-	1.04
spr1606	*pepF*	oligoendopeptidase F	-	-	-	1.07	-	-
spr1608	*prmA*	ribosomal protein L11 methyltransferase	-	-	1.03	-	-	1.06
spr1613	-	hypothetical protein	-	2.35	1.72	-	1.26	-
spr1629	-	transcriptional regulator	-	-	-	-	-	1.23
spr1670	*adhB*	zinc-containing alcohol dehydrogenase	-	-	-	-	1.40	1.51
spr1681	-	hypothetical protein	-	-	-	-	1.46	1.32
spr1688	-	hypothetical protein	-	-	-	1.56	1.37	-
spr1720	-	hypothetical protein	-	-	-	-	2.00	2.00
spr1723	*groES*	co-chaperonin GroES	-	-	1.27	1.03	1.78	2.13
spr1820	*nusG*	transcription antitermination protein NusG	-	-	1.00	-	1.02	-
spr1825	*gapA*	glyceraldehyde-3-phosphate dehydrogenase	-	-	-	1.13	-	-
spr1840	*yajC*	preprotein translocase subunit YajC	-	-	-	-	-	1.09
spr1866	*adh*	zinc-containing alcohol dehydrogenase	-	-	-	-	-	1.04
spr1920	*malD*	maltodextrin ABC transporter permease	-	-	-	1.58	-	-
spr1930	-	hypothetical protein	-	-	-	-	1.81	2.00
spr1945	*pcpA*	choline binding protein PcpA	-	-	-	-	-	1.22
spr1981	*dltB*	activated D-alanine transport protein	-	-	-	-	1.09	1.45
spr1982	*dltA*	D-alanine--poly(phosphoribitol) ligase subunit 1	-	-	-	-	1.00	1.34
spr1996	-	hypothetical protein	-	-	-	-	1.13	1.41
spr2011	-	ribosomal subunit interface protein	-	-	-	-	-	1.06
spr2045	*sphtra*	serine protease	-	-	-	-	-	1.43

Supplementary Table S2: Genes modulated by PEN in *S. pneumoniae* CCRI-21487 in the time-course transcriptomic design.

			Log$_2$FC (q- Value ≤ 0.05)					
CCRI-21487			No PEN			PEN at 1X MIC		
Entry no. in R6 genome database	Gene	Protein	8 min	18 min	28 min	8 min	18 min	28 min
**Downregulated**								
spr0030	-	hypothetical protein	-	-	-	-	-	-2.00
spr0041	*IS1167*	transposase	-	-	-	-1.27	-	-
spr0079	*transposase_A*	degenerative transposase	-	-	-1.06	-1.10	-	-
spr0225	-	hypothetical protein	-	-1.00	-	-1.42	-	-1.00
spr0382	*fabF*	3-oxoacyl-ACP synthase	-	-	-	-1.28	-	-
spr0383	*accB*	acetyl-CoA carboxylase biotin carboxyl carrier protein subunit	-1.07	-	-	-1.89	-1.26	-1.04
spr0384	*fabZ*	(3R)-hydroxymyristoyl-ACP dehydratase	-	-	-	-1.37	-	-
spr0385	*accC*	acetyl-CoA carboxylase biotin carboxylase subunit	-	-	-	-1.05	-	-
spr0386	*accD*	acetyl-CoA carboxylase subunit beta	-	-	-	-1.62	-	-
spr0387	*accA*	acetyl-CoA carboxylase subunit alpha	-	-	-	-1.34	-	-
spr0388	-	hypothetical protein	-	-	-	-1.22	-	-
spr0443	*glnR*	transcriptional repressor of the glutamine synthetase gene	-	-	-	-	-1.08	-2.32
spr0444	*glnA*	glutamine synthetase, type I	-	-	-1.27	-	-1.45	-2.82
spr0532	*glnP*	amino acid ABC transporter permease	-	-1.21	-1.45	-1.10	-	-2.10
spr0533	*glnP*	amino acid ABC transporter permease	-	-	-	-	-	-1.28
spr0534	*glnH*	amino acid ABC transporter amino acid-binding protein	-	-	-1.39	-	-	-3.09
spr0594	-	hypothetical protein	-	-	-1.20	-	-1.18	-
spr0644	*transposase_C*	transposase	-	-	-1.00	-1.42	-	-
spr0645	-	hypothetical protein	-	-2.22	-2.22	-2.81	-	-
spr0647	*pmi*	mannose-6-phosphate isomerase	-	-	-	-1.32	-	-
spr0650	-	hypothetical protein	-	-	-	-	-	-1.16
spr0683	-	hypothetical protein	-	-	-	-	-	-1.22
spr0722	*transposase_A*	transposase	-	-	-	-1.23	-	-
spr0740	*rpsT*	30S ribosomal protein S20	-	-	-	-1.02	-	-
spr0770	-	hypothetical protein	-	-1.84	-1.97	-1.73	-	-
spr0841	*IS1381*	tranposase	-	-	-	-	-	-1.58
spr0940	-	hypothetical protein	-	-	-	-	-	-1.00
spr1009	*IS1167*	transposase	-	-1.81	-2.81	-1.22	-	-
spr1020	*hlpA*	DNA-binding protein HU	-	-	-1.20	-1.03	-	-
spr1038	*transposase_A*	transposase	-	-1.17	-1.58	-1.49	-	-
spr1064	*nrdH*	glutaredoxin-like protein	-	-	-1.13	-	-	-
spr1079	-	hypothetical protein	-	-	-	-1.00	-	-
spr1120	*glnP*	amino acid ABC transporter substrate-binding protein	-	-	-	-	-	-1.45
spr1121	*glnQ*	amino acid ABC transporter ATP-binding protein	-	-	-	-	-1.10	-2.23
spr1467	*rpsO*	30S ribosomal protein S15	-	-1.04	-1.00	-	-1.02	-
spr1476	-	hypothetical protein	-	-	-	-	-	-1.58
spr1538	*axe1*	acetyl xylan esterase	-1.00	-1.57	-1.54	-	-1.09	-
spr1545	-	hypothetical protein	-1.00	-1.00	-	-1.00	-	-
spr1621	*scrR*	sucrose operon repressor	-	-1.00	-	-	-	-1.00
spr1837	*adhE*	bifunctional acetaldehyde-CoA/alcohol dehydrogenase	-	-	-	-1.31	-	-
spr1866	*adh*	zinc-containing alcohol dehydrogenase	-	1.11	-	-	1.13	1.25
spr1891	*IS1381*	tranposase	-	-1.32	-1.32	-1.32	-	-1.32
spr1925	-	hypothetical protein	-	-1.18	-1.56	-1.32	-	-
spr2022	*mreD*	rod shpae-determining protein MreD	-	-	-	-	-	-1.02
**Upregulated**								
spr0096	-	LysM domain-containing protein	-	-	-	-	-	1.28
spr0230	*PTS-EIIB*	PTS system transporter subunit IIB	-	-	-	1.17	-	-
spr0247	*pulA*	alkaline amylopullulanase	-	1.22	1.22	-	1.32	1.50
spr0260	*manM*	PTS system mannose-specific transporter subunit IIC	1.50	1.42	1.71	1.15	1.35	-
spr0307	*clpL*	ATP-dependent protease ATP-binding subunit	-	-	-	-	-	1.13
spr0415	*pfl*	formate acetyltransferase	-	-	-	-	-	1.03
spr0453	*hrcA*	heat-inducible transcription repressor	-	-	-	-	-	1.21
spr0455	*dnaK*	molecular chaperone DnaK	-	-	-	-	-	1.01
spr0456	*dnaJ*	molecular chaperone DnaJ	-	-	-	-	-	1.15
spr0562	*PTS-EII*	PTS system transporter subunit IIA	-	1.32	1.81	-	-	1.58
spr0564	*PTS-EII*	PTS system transporter subunit IIC	1.00	-	1.74	1.00	1.22	1.74
spr0565	*bgaA*	beta-galactosidase	1.08	1.08	1.15	-	1.15	-
spr0656	*clpP*	ATP-dependent Clp protease proteolytic subunit	-	-	-	-	-	1.16
spr0778	*fruR*	lactose PTS system repressor	1.72	1.84	1.80	1.63	1.86	1.70
spr0779	*fruB*	1-phosphofructokinase	1.76	1.90	1.75	1.67	1.96	1.56
spr0780	*fruA*	PTS system fructose specific transporter subunit IIABC	1.40	1.16	1.00	1.07	1.25	-
spr1046	*xerS*	site-specific tyrosine recombinase XerS	-	-	1.46	-	-	1.81

Supplementary Table S2: Genes modulated by PEN in *S. pneumoniae* CCRI-21487 in the time-course transcriptomic design *(Continued).*

			Log_2FC (*q*- Value ≤ 0.05)					
CCRI-21487			No PEN			PEN at 1X MIC		
Entry no. in R6 genome database	Gene	Protein	8 min	18 min	28 min	8 min	18 min	28 min
**Upregulated**								
spr1073	*lacD*	tagatose 1,6-diphosphate aldolase	1.49	1.32	-	1.67	1.49	1.52
spr1074	*lacC*	tagatose-6-phosphate kinase	1.24	-	-	1.13	-	-
spr1075	*lacB*	galactose-6-phosphate isomerase subunit LacB	1.49	-	-	2.07	1.85	1.93
spr1076	*lacA*	galactose-6-phosphate isomerase subunit LacA	1.13	-	-	1.54	-	-
spr1272	*nagB*	glucosamine-6-phosphate isomerase	-	-	-	-	-	1.44
spr1420	-	hypothetical protein	-	-	-	1.19	-	-
spr1455	*phnA*	hypothetical protein	-	-	1.24	-	-	1.14
spr1523	-	hypothetical protein	1.27	1.58	1.88	-	1.53	1.66
spr1530	-	hypothetical protein	2.16	2.50	2.66	1.74	2.32	2.22
spr1531	*nanB*	neuraminidase B	2.17	3.09	3.00	2.17	2.91	2.58
spr1532	*ABC-MSP*	ABC transporter permease	2.00	2.58	2.32	1.58	2.32	2.00
spr1623	-	hypothetical protein	1.27	-	-	1.04	-	-
spr1629	-	transcriptional regulator	-	-	-	-	-	1.29
spr1685	*fatC*	iron-compound ABC transporter permease	1.07	-	-	-	1.02	-
spr1686	*fecE*	iron-compound ABC transporter ATP-binding protein	1.17	-	-	1.07	-	-
spr1687	*fatB*	iron-compound ABC transporter substrate-binding protein	1.27	1.07	-	1.02	1.06	-
spr1723	*groES*	co-chaperonin GroES	-	-	-	-	-	1.48
spr1812a	-	hypothetical protein	-	-	-	1.46	-	-
spr1866	*adh*	zinc-containing alcohol dehydrogenase	-	1.11	-	-	1.13	1.25
spr1895	*pstS*	phosphate ABC transporter substrate+B766-binding protein	-	1.81	-	-	1.81	1.86
spr1897	*pstA*	phosphate ABC transporter permease	-	-	-	-	1.58	-
spr1898	*pstB*	phosphate transporter ATP-binding protein	-	1.76	-	-	1.82	1.66
spr1899	*phoU*	phosphate transporter PhoU	-	1.78	-	-	1.78	-
spr1988	*glpF*	glycerol uptake facilitator protein	-	-	-	1.58	-	-
spr1991	*glpK*	glycerol kinase	-	-	-	1.14	-	-
spr2003	-	ABC transporter substrate-binding protein	-	1.38	1.40	-	1.30	1.16
spr2011	-	ribosomal subunit interface protein	-	1.33	1.27	1.31	1.55	2.15

Supplementary Table S3: Genes modulated in a common fashion by PEN in *S. pneumoniae* CCRI-8970 and CCRI-21487.

				Log_2FC (*q*- Value ≤ 0.05)					
				**CCRI-8970**			**CCRI-21487**		
**Entry no. in R6 genome database**	**Gene Symbol**	**Gene description**	**Condition**	**T1/T0**	**T2/T0**	**T3/T0**	**T1/T0**	**T2/T0**	**T3/T0**
**Down-regulated**									
spr0443	*glnR*	transcriptional repressor of the glutamine synthetase gene	NO PEN	-	-2.53	-2.35	-	-	-
			PEN 1X MIC	-2.08	-3.02	-2.93	-	-1.08	-2.32
spr0444	*glnA*	glutamine synthetase, type I	NO PEN	-1.42	-	-	-	-	-1.27
			PEN 1X MIC	-1.75	-1.45	-1.65	-	-1.45	-2.82
spr0532	*glnP*	amino acid ABC transporter permease	NO PEN	-	-	-1.20	-	-1.21	-1.45
			PEN 1X MIC	-1.92	-1.82	-1.82	-1.10	-	-2.10
spr0533	*glnP*	amino acid ABC transporter permease	NO PEN	-	-1.46	-1.75	-	-	-
			PEN 1X MIC	-2.00	-2.30	-2.42	-	-	-1.28
spr0534	*glnH*	amino acid ABC transporter amino acid-binding protein	NO PEN	-1.98		-1.44	-	-	-1.39
			PEN 1X MIC	-2.40	-2.48	-2.76	-	-	-3.09
spr1120	*glnP*	amino acid ABC transporter substrate-binding protein	NO PEN	-	-	-	-	-	-
			PEN 1X MIC	-1.30	-1.69	-2.04	-	-	-1.45
spr1121	*glnQ*	amino acid ABC transporter ATP-binding protein	NO PEN	-1.02	-1.55	-1.80	-	-	-
			PEN 1X MIC	-2.05	-2.63	-2.96	-	-1.10	-2.23
**up-regulated**									
spr0307	*clpL*	ATP-dependent protease ATP-binding subunit	NO PEN	-	1.33	1.81	-	-	-
			PEN 1X MIC	1.30	2.03	2.22	-	-	1.13
spr0455	*dnaK*	molecular chaperone DnaK	NO PEN	-	1.50	1.50	-	-	-
			PEN 1X MIC	1.33	1.71	1.75	-	-	1.01
spr0456	*dnaJ*	molecular chaperone DnaJ	NO PEN	-	2.01	1.86	-	-	-
			PEN 1X MIC	1.80	1.97	2.10	-	-	1.15
spr0656	*clpP*	ATP-dependent Clp protease proteolytic subunit	NO PEN	-	-	-	-	-	-
			PEN 1X MIC	-	1.21	1.49	-	-	1.16
spr1629	-	transcriptional regulator	NO PEN	-	-	-	-	-	-
			PEN 1X MIC	-	-	1.23	-	-	1.29
spr1723	*groES*	co-chaperonin GroES	NO PEN	-	-	1.27	-	-	-
			PEN 1X MIC	1.03	1.78	2.13	-	-	1.48
spr1866	*adh*	zinc-containing alcohol dehydrogenase	NO PEN	-	-	-	-	1.17	-
			PEN 1X MIC	-	-	1.04	-	1.13	1.25
spr2011	-	ribosomal subunit interface protein	NO PEN	-	-	-	-	1.33	1.27
			PEN 1X MIC	-	-	1.06	1.31	1.55	2.15

Supplementary Table S4: List of primers used in this study.

**Primers**	**Sequence (5'-->3')**
**For qRT-PCR**	
Frd spr0443 *glnR*	CATGGATCGTCTGCTTGAAA
Rv spr0443 *glnR*	CTTGGATTTCGCTTCACGTT
Frd spr0444 *glnA*	TCTTGAGTTGCGTTCAGTGG
Rv spr0444 *glnA*	TTCGATAGGAGCTGGTGCTT
Frd spr1120 *glnP*	CTGGGGAATTCCAAACTTCA
Rv spr1120 *glnP*	ATTGAGTGAGAGGGCAATGG
Frd spr1121 *glnQ*	CTCGTGGCCTAGCAATGAAT
Rv spr1121 *glnQ*	ACGTCTCCAACCATCTCAGG
Frd spr0534 *glnH*	CTATCACCCAACGCCTGATT
Rv spr0534 *glnH*	CTGGGTAGGAACCAAGTTCG

<u>LC-MS analysis</u>

Chemicals

All chemicals and standards were HPLC grade. Acetonitrile (A955-4L); formic acid (A117-50) were purchased from Fisher Scientific, and hydrochloric acid (HCl, 258148); L-glutamine (G3126); glutamate (G1251) from Sigma Aldrich. Deuterated DL-glutamate (2.3.3.4.4-D5,97%, cat no. DLM-357-0) and deuterated L-Glutamine (2,3,3,4,4-D5, 97%, cat no. DLM-1826-0.1) were obtained from Cambridge Isotope Laboratories, Inc. Ultrapure water was produced by a Millipore SuperQ system for mobile phase and reagent dilution.

Internal standard preparation

Deuterated standards stock solutions were prepared in 1N HCl at 1 mg/mL. A mix of two deuterated stock solutions for dGlu and dGln was diluted at 100 µg/mL for calibration curves. A second mixture with dGlu at 20 µg/mL and dGln at 12 µg/mL prepared in water was also prepared for spiking in the extraction solution.

Working mixture and calibration curves

A mix of two deuterated internal standards (IS) DL-glutamate (dGlu) and L-Glutamine (dGln) was spiked during cells lysis to a final concentration of 500 ng/mL and 300 ng/mL respectively. Lyophilised extracts with IS were resuspended in 150 µL of 50:50 (mobile phase A: mobile phase B) and sonicated 5 min at 15°C. Samples were then filtrated with a 13 mm 0.22 µM PVDF syringe filter (Fisherbrand, no 09-720-3) before injection in UPLC Acquity system. For calibration curves, 15 extraction tubes of T0 wild type *S.*

pneumoniae were pooled and used as matrix dilution. The linear regression was eight-points serial dilutions of a mixture of dGlu and dGln (0; 23.4; 46.8; 93.7; 187.5; 375; 750; 1500 ng/mL) in the matrix. Linearity was determined by the squared correlation coefficient (R^2). Quantification was expressed as the peak-area ratio of deuterated molecule (dGlu or dGln) on its respective endogenous signal (Glu or Gln). As for analytes, quantification was expressed as peak-area ratio of endogenous signal against deuterated IS. TargetLynx software from Waters Corp. was used to assess peak integration. The method monitors the fragment peak area of the molecule of interest to a specified retention time. Data were then exported to Microsoft Excel to allow linearity, standard deviation and quantification measurements.

Method validation

Quality control (QC) was injected three times for curve validation. Concentration of a dGlu/dGln QC mix was tested at low (80 ng/mL), medium (160 ng/mL) and high (320 ng/mL) concentrations. Validation curves were accepted when the QC concentration did not exceed 15% of the standard deviation of expected value. The matrix effect was compensated by performing curves and IS dilutions in a pool of matrix from *S. pneumoniae* untreated samples. Three technical and biological replicates allowed reproducibility and variability measurements. Samples were randomly injected and carry-over was evaluated by several randomized blank injection.

Supplementary Table S5: Compounds parameters and linearity

Compounds	*S.pneumoniae strains*	Retention time (min)	Formula	Precursor ions (m/z)	Product ions (m/z)	Regression equation	R^2
Endogenous glutamine	R6	5,18	C5H10N2O3	146.0686	130.0500	y = 63,676x + 3,7381	0,9992
	CCRI-8970	5,18	C5H10N2O3	146.0686	130.0500	y = 1075,3x - 3,087	0,9994
	CCRI-21487	5,18	C5H10N2O3	146.0686	130.0500	y = 108,93x + 8,2567	0,9994
Endogenous glutamate	R6	4,84	C5H9NO4	147.0526	130.0500	y = 616,74x + 12,99	0,9991
	CCRI-8970	4,84	C5H9NO4	147.0526	130.0500	y = 2252,4x + 3,9258	0,9997
	CCRI-21487	4,84	C5H9NO4	147.0526	130.0500	y = 1770,4x + 15,827	0,9983
Deuterated glutamine	R6	5,18	C5D5H5N2O3	151.1077	135.0844	y = 63,676x + 3,7381	0,9992
	CCRI-8970	5,18	C5D5H5N2O3	151.1077	135.0844	y = 1075,3x - 3,087	0,9994
	CCRI-21487	5,18	C5D5H5N2O3	151.1077	135.0844	y = 108,93x + 8,2567	0,9994
Deuterated glutamate	R6	4,84	C5D5H4NO4	152.0901	135.0844	y = 616,74x + 12,99	0,9991
	CCRI-8970	4,84	C5D5H4NO4	152.0901	135.0844	y = 2252,4x + 3,9258	0,9997
	CCRI-21487	4,84	C5D5H4NO4	152.0901	135.0844	y = 1770,4x + 15,827	0,9983

Supplementary Table S6: Compounds MS settings

Tune page	Cone voltage	3
	Polarity	ESI+
	Da Range	50-600
	Scan time	0.1 sec Continium
	Capillary (kV)	2.75
	Sampling cone	50
	Source offset	80
	Source temperature	120
	Desolvatation temperature	325
	Cone gas	10
	Desolvatation gas	900
	Nebuliser	6
Lock mass solution	Leucine-Enkephaline	200 pg/µL
Lock mass flow rate		20 µL/ min
Transfer CE ramp (V)	Endogenous glutamine	3-5
	Endogenous glutamate	3-5
	Deuterated glutamine	3-5
	Deuterated glutamate	5-7
